# Strength performance of hexagonal, hollow, and perforated GFRP-Reinforced concrete units: experimental, numerical, and AI-based investigations

**DOI:** 10.1038/s41598-026-49173-z

**Published:** 2026-04-21

**Authors:** Mohammadamin Mirdarsoltany, Nima Khodadadi, Antonio Nanni

**Affiliations:** 1 Department of Civil and Architectural Engineering, University of Miami, Coral Gables, FL 33146 USA; 2 Department of Civil and Environmental Engineering, Pacific Earthquake Engineering Research (PEER) Center, University of California, Berkeley, CA USA

**Keywords:** Artificial Intelligence, Finite Element Modeling, Hexagonal, Hollow, Glass Fiber-Reinforced Polymer, Differential Evolutionary Gene Expression Programming, Engineering, Materials science

## Abstract

This study investigates the structural performance of hexagonal, hollow, perforated SEAHIVE^®^ concrete units reinforced with GFRP bars under transverse compression and flexural loading. An integrated experimental–numerical–artificial intelligence framework was developed to establish structural capacity and derive design-oriented predictive equations. Nonlinear finite element models were implemented in ABAQUS using the Concrete Damage Plasticity (CDP) formulation and validated against experimental load–displacement behavior and crack patterns. Parametric analysis demonstrated that element thickness is the dominant factor governing compressive capacity, with increases from 100 mm to 180 mm resulting in over 280% improvement. Increasing concrete compressive strength from 20 MPa to 40 MPa enhanced transverse compression and flexural capacities by up to 60% and 66%, respectively, while larger perforation diameters reduced capacity due to stress concentration effects. To improve practical applicability, Differential Evolution Chromosomal Gene Expression Programming (DEC-GEP) was employed to develop explicit predictive equations. The symbolic regression models achieved coefficients of determination (R²) of 0.948 for transverse compression and 0.921 for flexure, providing accurate and interpretable tools for preliminary design. The proposed framework offers a validated approach for evaluating perforated coastal concrete systems and supports their optimized application in sustainable shoreline infrastructure.

## Introduction

 Coastal regions face escalating challenges due to climate change and human activities, raising critical questions about their future habitability and economic viability. Beyond the threats to human activities, coastal ecosystems such as mangroves, coral reefs, seagrass meadows, and salt marshes, which support numerous communities, are at risk. Rising sea levels compound these issues, presenting a growing concern for preserving both natural habitats and infrastructure^1–3^. In addition, nearly 40% of the global population lives near coastlines, making them highly vulnerable to extreme weather events such as hurricanes and tropical storms^4^. These natural disasters cause significant economic losses and have severe social and psychological impacts^4–7^. A recent National Centers for Environmental Information report estimated that the economic cost of catastrophic weather events, including hurricanes, in the United States exceeded $2 trillion, with flooding accounting for a substantial portion^8^. Addressing these challenges requires innovative approaches to mitigate the risks and reduce economic losses in coastal areas^9^.

Traditional shoreline protection measures, such as seawalls, have been extensively used but often fail to provide effective and sustainable solutions^10,11^. Conventional seawalls may fail to dissipate wave energy efficiently and can increase sediment suspension, exacerbating shoreline erosion^12^. Furthermore, these structures are not environmentally friendly, with studies showing they reduce biodiversity by 23% and organism populations by 45%^10,13,14^. To address this issue, efforts have been made to design innovative structures that provide effective, eco-friendly coastal protection while fostering a hospitable environment for marine life. Among these, SEAHIVE^®^ units stand out as a novel solution^10^. SEAHIVE^®^ units are modular, hexagonal, hollow, and perforated concrete structures designed for both hydraulic and structural performance. However, when it comes to the reinforcement, coastal construction using reinforced concrete (RC) with carbon steel reinforcement also faces limitations due to the steel’s susceptibility to corrosion, especially in areas exposed to tidal and wave action, leading to reduced service life^15^. To address these issues, glass fiber-reinforced polymer (GFRP) bars, which are corrosion-resistant, have been proposed as an alternative for RC structures in coastal applications^16,17^. However, structural design guidance for innovative perforated geometries remains limited^18^.

Proposed alternative, with its geometric features, enables wave-energy dissipation, while perforations promote ecological connectivity. Moreover, internally reinforced with GFRP bars, they offer corrosion resistance for long-term marine applications. These hollow hexagonal RC units, featuring perforations, demonstrate excellent wave-energy dissipation properties and are more environmentally sustainable than conventional coastal structures^12,14^. Beyond their application in shoreline protection, SEAHIVE^®^ technology holds potential as a countermeasure against bridge pier scour, one of the leading causes of bridge failure^19^. With perforations that can alter hydraulic flow dynamics, these units may help mitigate contraction scour around pile caps and within interstitial spaces between piles^16,20^.

While previous research has extensively studied the wave-energy dissipation capabilities of SEAHIVE^®^ units, there is a lack of data on their structural performance under different fabrication methods and loading conditions. Existing studies on beams with web openings provide some insights^21–23^, but the unique geometry and hollow cross-section of SEAHIVE^®^ units introduce additional complexities. Research on solid beams with openings has shown that factors such as opening size, shape, and placement significantly influence performance, with circular openings generally outperforming other shapes in terms of structural efficiency^24–27^. However, the flexural and transverse compressive behavior of perforated hollow hexagonal units, such as SEAHIVE^®^, remains largely unexplored. These previous investigations primarily focused on prismatic rectangular members subjected to conventional flexural or shear loading, and their findings cannot be directly extended to multi-faceted hollow coastal units with distributed perforations and inclined load paths. Consequently, no validated structural capacity framework currently exists for hexagonal, hollow, perforated concrete units reinforced with GFRP bars.

SEAHIVE^®^ units, internally reinforced with GFRP bars, present a lightweight yet durable option for structural applications in marine and terrestrial environments. However, their intricate design poses challenges related to stress distribution, crack propagation, and load-bearing capacity^19^. The presence of multiple perforations and hollow sections leads to nonlinear stress redistribution and localized crack concentration, which cannot be accurately captured by classical beam theory or simplified analytical approaches. Therefore, advanced nonlinear finite element modeling (FEM) techniques are required to simulate cracking behavior, stiffness degradation, and failure mechanisms with greater reliability. To address these challenges, this study focuses on a comprehensive parametric analysis to evaluate the structural performance of SEAHIVE^®^ units. By systematically varying parameters such as concrete strength, unit thickness, and perforation diameter using FEM in ABAQUS, the research aims to optimize the design and enhance the performance of these units. Unlike prior analytical or partially validated numerical studies, the present work employs experimentally calibrated material models and validates nonlinear FEM predictions against laboratory load–displacement responses and observed crack patterns. This validation process enhances the predictive accuracy and reliability of the numerical framework before conducting an extensive parametric investigation. Furthermore, the systematic evaluation of interacting geometric and material parameters establishes a consistent numerical database that has not been previously reported for such perforated hollow systems.

Another goal of this research is to provide engineers with practical equations using artificial intelligence (AI) to facilitate the adoption of SEAHIVE^®^ technology^28,29^. Engineers often hesitate to adopt new solutions without reliable, accessible design resources, as such equations are crucial for field implementation. This study bridges that gap by developing a parametric design framework that enables engineers to confidently incorporate SEAHIVE^®^ units into various structural applications while ensuring safety and efficiency (Note: The FEM and AI are only valid for the given overall dimensions of the X-section).

This paper builds on previously published experimental results to present a detailed parametric study of SEAHIVE^®^ units^30^. The findings contribute to the development of lightweight, sustainable, and efficient structural systems by analyzing the effects of key parameters on transverse compressive and flexural performance. The study advances the understanding of SEAHIVE^®^ units and provides valuable insights for their practical design and application. Unlike prior studies on wave-dissipating units, this research addresses the structural capacity of perforated, hollow, and GFRP-reinforced units. The integration of FEM and AI-based modeling for SEAHIVE^®^ structural performance is a novel contribution.

Accordingly, this research fills four primary gaps in the existing literature: (1) absence of validated structural performance data for perforated hollow hexagonal units reinforced with GFRP bars; (2) limited understanding of crack propagation mechanisms under transverse compression and flexure; (3) lack of a systematic parametric framework quantifying the interaction between material and geometric variables; and (4) absence of explicit, design-oriented predictive equations for structural capacity estimation. The proposed integrated experimental–FEM–AI framework provides a foundation for future optimization studies, expanded geometries, and broader applications across coastal infrastructure.

## Test specimens

Four SEAHIVE^®^ specimens were evaluated: two under transverse compression and two under flexural loading. Transverse compression specimens measured 0.91 m in length, while flexural specimens were 1.83 m long. The hexagonal cross-sections featured circular perforations (200 mm diameter) and reinforcement with M15 and M10 GFRP bars. Detailed configurations and preparation methods are presented in a publication by Mirdarsoltany et al.^30^.

## Materials

### GFRP and concrete characterization

The tensile properties of M10 and M15 GFRP bars were determined according to ASTM D7205. M15 bars showed a reduction in tensile strength compared to M10 due to the shear-lag effect as summarized in Table 1.

**Table 1:** Material properties of GFRP bars^30^.

Designation	Elastic modulus (MPa)	Ultimate tensile strength (f_fu_)(MPa)	Ultimate strain	Concrete clear cover (mm)
M15	60,790	991	0.0163	63.5
M10	61,600	1274	0.021	58.5

Concrete compressive strength tests conducted in accordance with ASTM C39 yielded an average strength of 20 MPa. Table 2 outlines key material properties, with further mixed design details found in^30^.

**Table 2:** Material properties of concrete.

Designation	Elastic modulus (MPa)	Ultimate strain	Concrete strength (MPa)
Concrete	24,870*	0.003**	20.0

Notes: *= value derived from code provisions; **=design value from code.

## Test setup and instrumentation

### Half unit under transverse compression

**Fig. 1:**
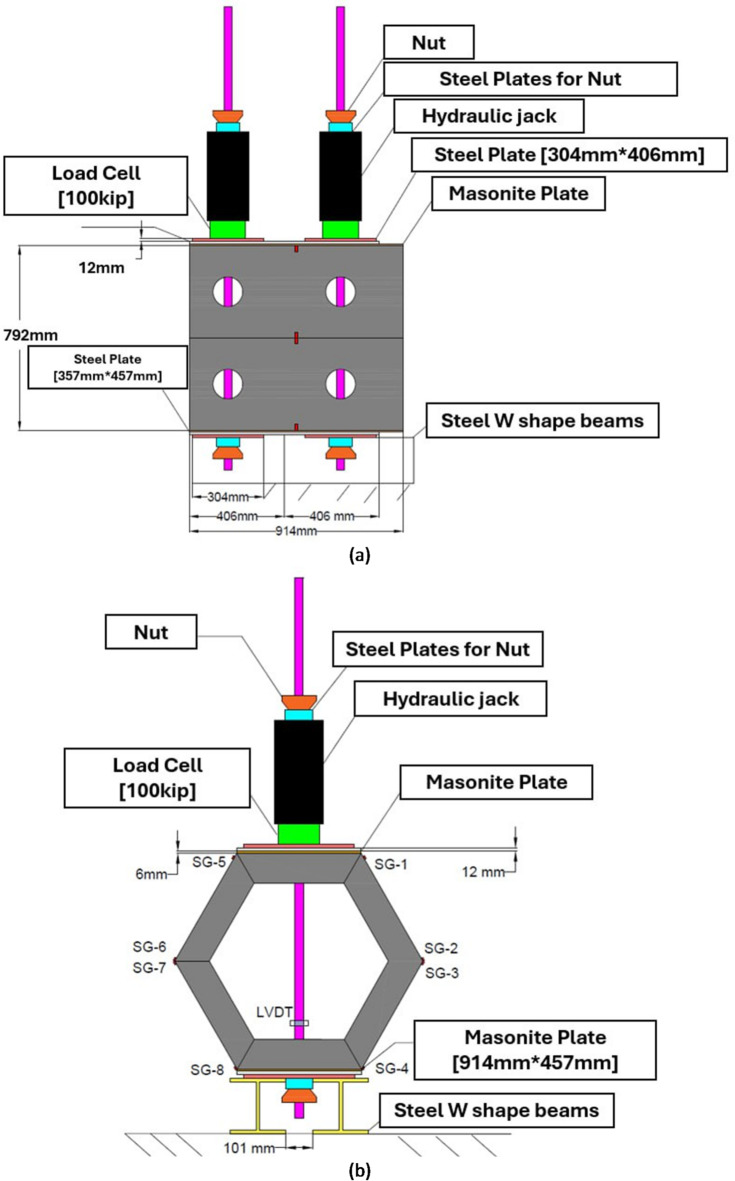
Transverse compression test setup: (**a**) side and (**b**) cross-sectional views.

Transverse compression tests on 0.91 m specimens were conducted using hydraulic jacks, with uniform load distribution ensured through masonite sheets and steel plates. The setup is illustrated in Fig. 1(a–b).

### Unit under flexure

**Fig. 2:**
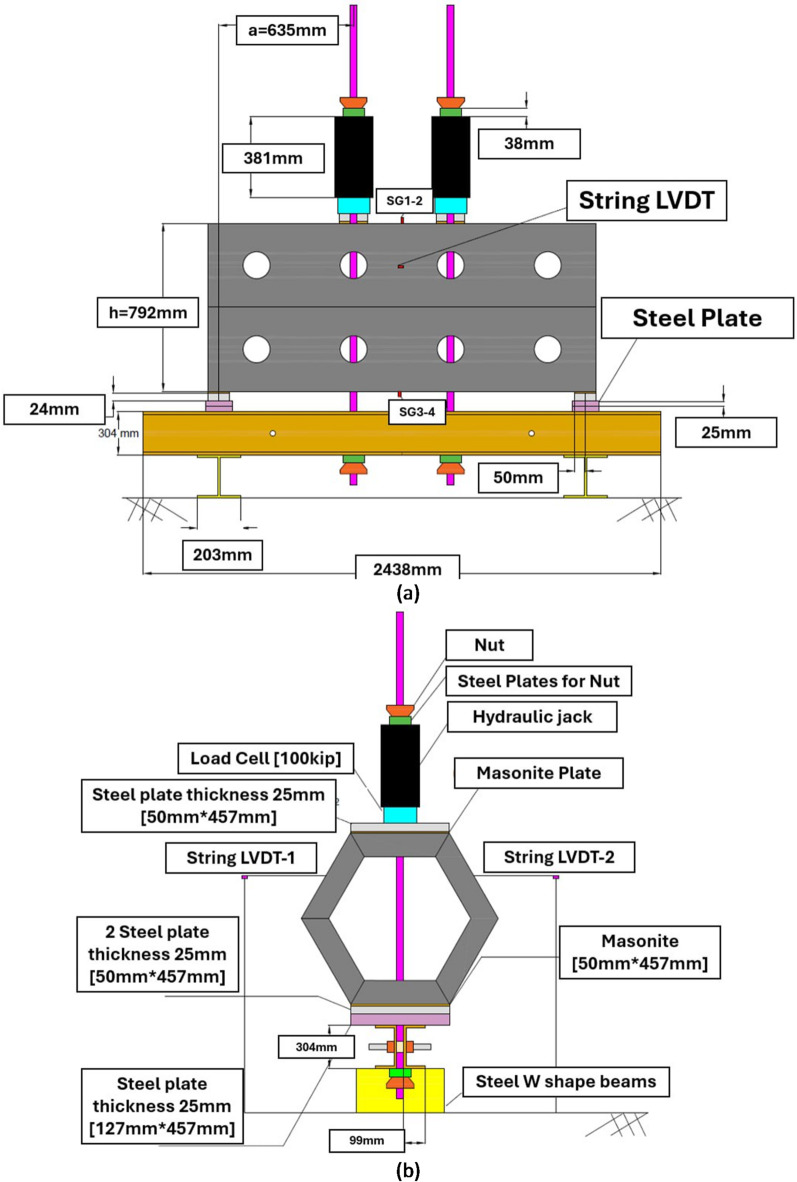
Flexural test setup: (**a**) side and (**b**) cross-sectional views.

Flexural tests were conducted in a four-point bending configuration. Specimens were classified as deep beams with an a/h ratio of 0.8. Hydraulic jacks applied loads, and strain gauges measured mid-span strain distributions. See Fig. 2 (a–b).

## Loading protocol

Both transverse compression and four-point flexural tests utilized quasi-static load protocols. The first specimen was subjected to a continuously increasing load in each test type until failure to determine its ultimate load capacity. The second specimen underwent incremental cyclic loading and unloading to study crack development and deformation recovery. Two load-unload cycles were performed, with peak loads set at roughly one-third and two-thirds of the ultimate capacity determined from the monotonic test, followed by a final cycle leading to failure.

## Results and discussion

This section presents the experimental findings, including crack patterns and load-displacement behavior observed in both transverse compression and bending tests. Key parameters such as the initial cracking load, ultimate load, and maximum displacement or mid-span Deflection were documented.

### Transverse compression test results

**Fig. 3:**
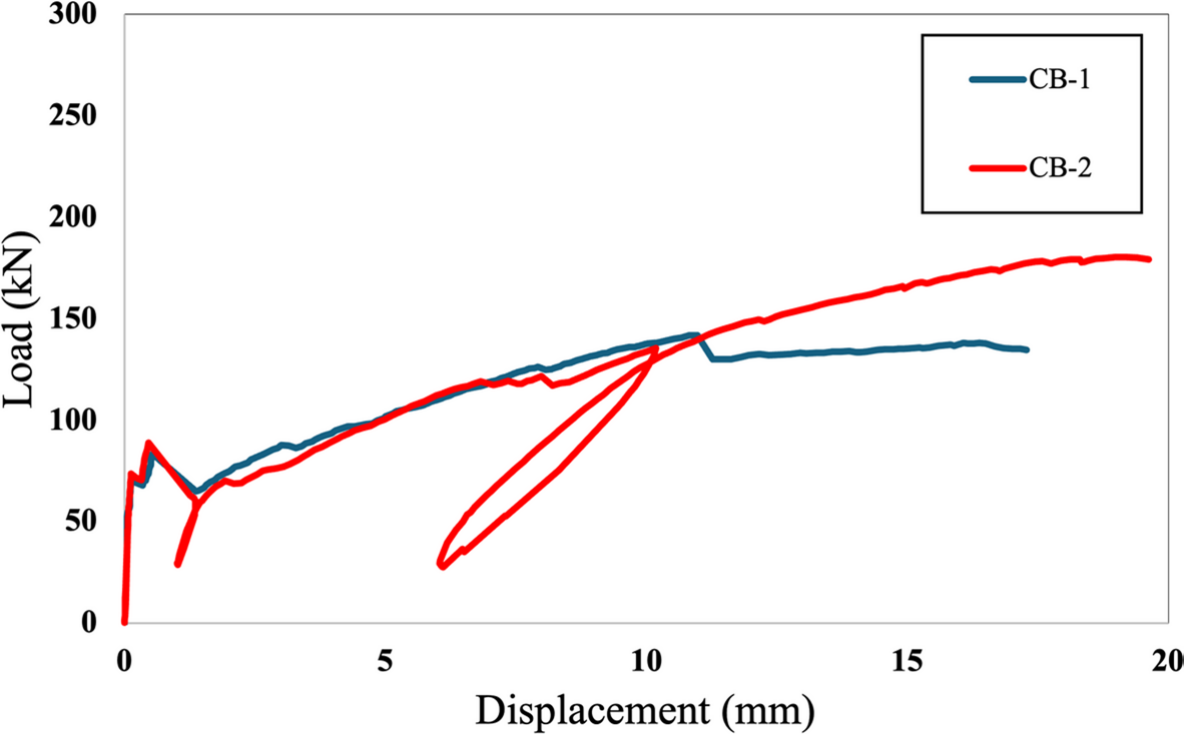
Load-displacement curve of CB-1 and CB-2.

The load-displacement curves for the transverse compressive tests of specimens CB-1 and CB-2 illustrate their structural response under uniform axial transverse compression (Fig. 3). Both specimens demonstrate an initial linear elastic behavior, indicating stiffness prior to the onset of cracking. For CB-1, the curve shows a sudden drop in load at a displacement of around 5 mm, corresponding to the formation of the first crack. Following this, the specimen continues to sustain load with reduced stiffness until reaching its ultimate load capacity of approximately 143 kN, after which failure occurs. In the case of CB-2, tested under cyclic loading, the curve exhibits progressive load-unload cycles with increasing displacements. The first load cycle reaches about one-third of the ultimate load, and the second load cycle peaks at two-thirds of the ultimate load, reflecting the quasi-static loading protocol.

### Flexure test results

**Fig. 4:**
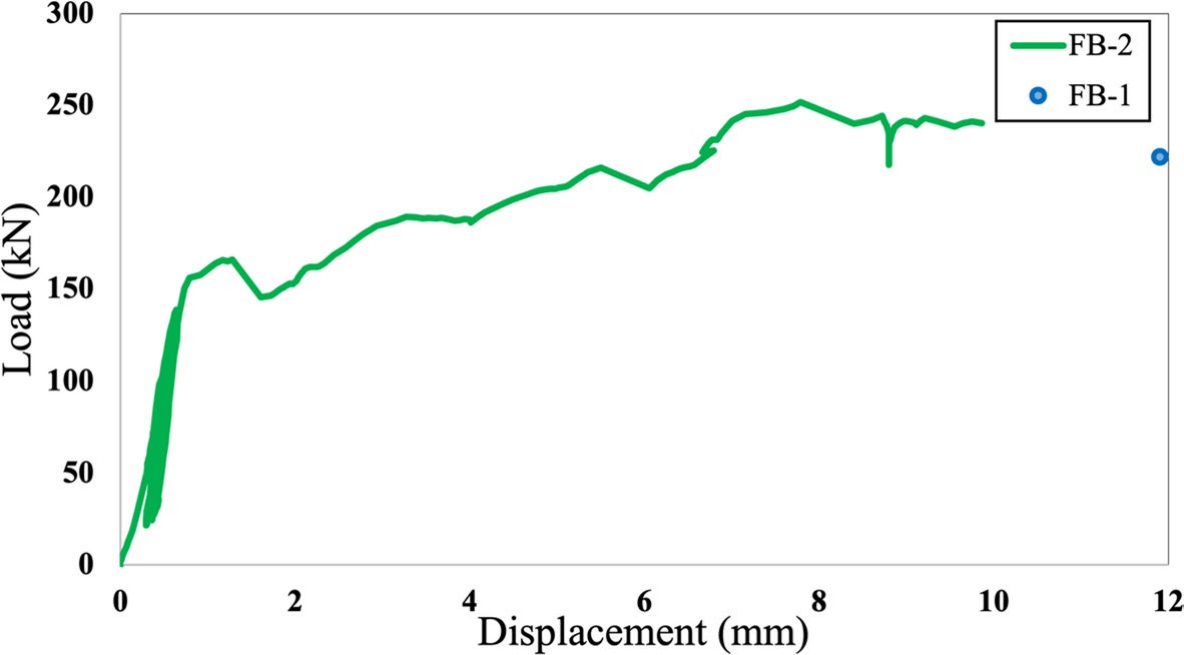
Load-displacement curve of FB-2.

Due to a data logger failure, strain gauge, and LVDT measurements for FB-1 were unavailable. However, the hydraulic pressure gauge indicated cracking and failure loads of approximately 90 kN and 220 kN, respectively. For FB-2, the load-displacement curve (Fig. 4) demonstrates that the first significant load drop, marking the initial crack formation, occurred at 156 kN (Table 3). The horizontal axis represents the average displacement from two LVDTs, while the vertical axis shows the total applied load measured by two load cells.

**Table 3:** Experimental outcomes.

Specimen ID	First cracking load (kN)	Ultimate load (kN)	Maximum Deflection (mm)
CB-1	73	143	17
CB-2	79	179	19
FB-1	89	222	12
FB-2	156	250	10

## Parametric study

### Specimens under transverse compression

**Fig. 5:**
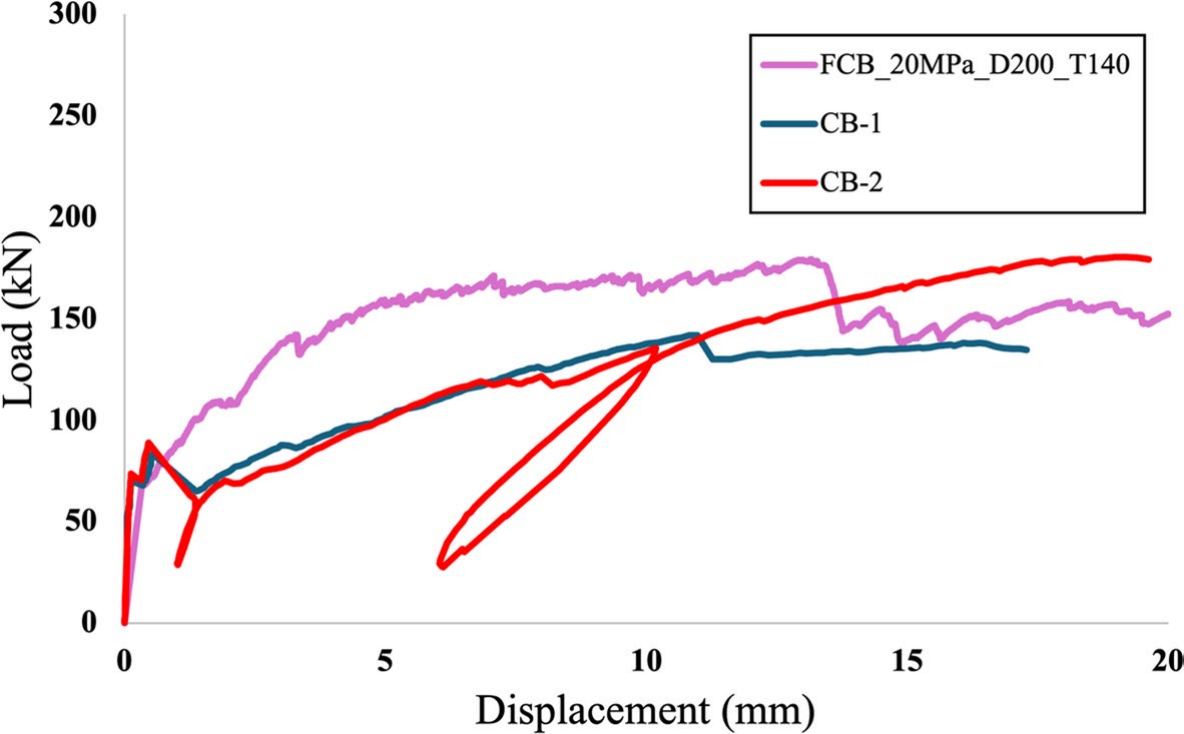
Load displacement curve obtained from FEM and experiment.

FEM was employed using ABAQUS software to evaluate the element’s performance under transverse compression. The purpose of developing the FEM model was to simulate crack propagation with special attention to the inclined leg. Material properties were defined based on experimental data, and the concrete damage plasticity (CDP) model was used to represent concrete with a compressive strength of 20 MPa. A static general procedure was adopted for the analysis. Furthermore, displacement was applied rather than the load directly to the top of the sample. This approach helps minimize potential errors during the analysis. Figure 5 shows the load-displacement curves obtained from the ABAQUS software and experiments. As depicted in the figure, the first crack observed in the numerical model aligns with the experimental results. However, the analytical prediction indicates a higher load level after the initial crack formation, up to approximately 15 mm of deflection. This discrepancy may be due to imperfections in the test specimens during loading. Beyond 15 mm of Deflection, up to the point of failure, the numerical model again aligns closely with the experimental results.

**Fig. 6:**
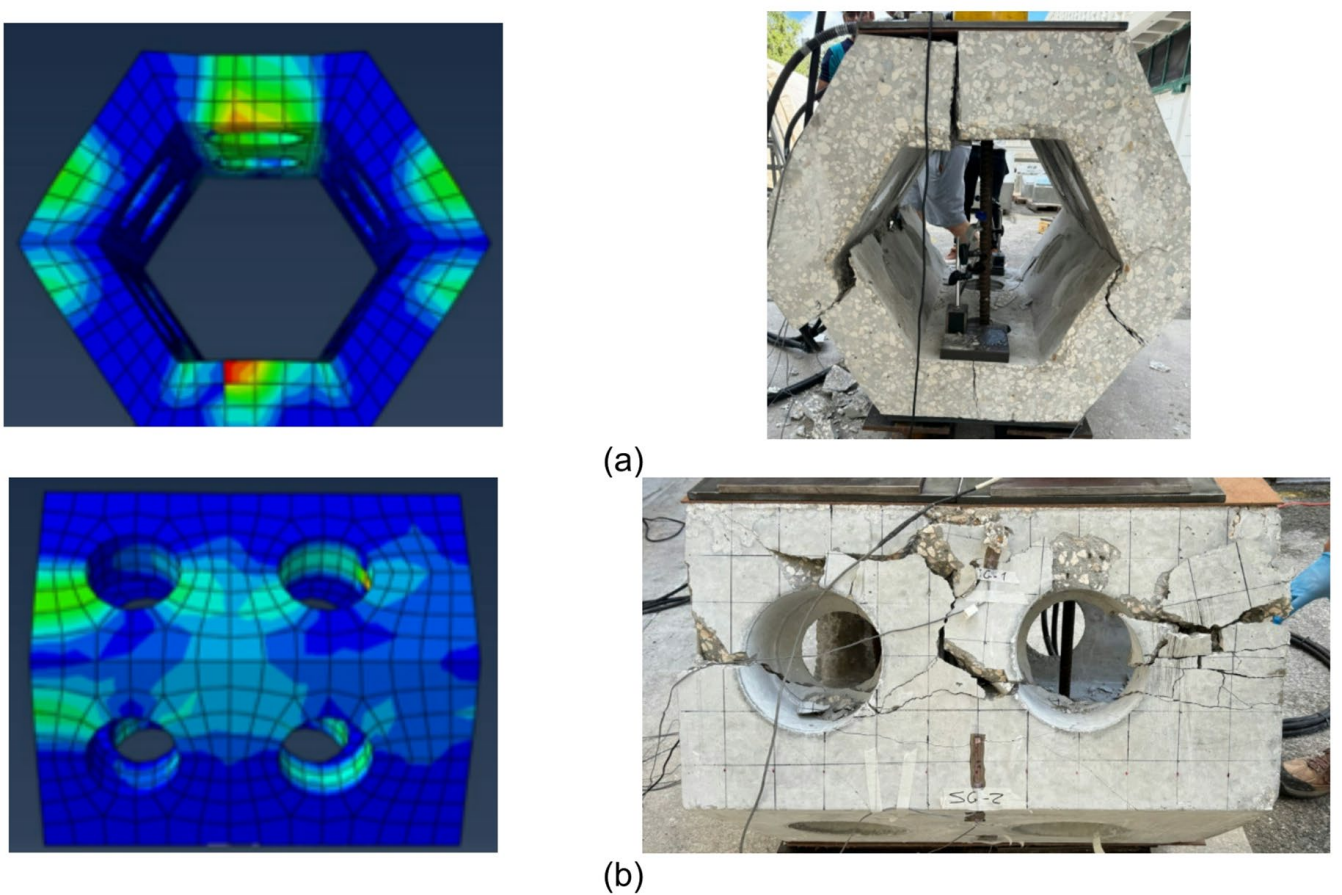
Comparing the crack propagation in the FEM model and experiment (**a**) cross-section and (**b**) side of the element.

Figure 6 shows the PEMAG (percentage magnitude of plastic strain energy) contour, which illustrates the magnitude of plastic strain, and compares crack propagation between experimental results and numerical models. Derived from finite element simulations, it represents the percentage of plastic strain energy in a material, highlighting regions of tensile stress exceeding the elastic limit and leading to plastic deformation. These contours, expressed as percentages, identify areas with higher plastic strain energy, offering insights into material behavior and stress distribution. This scalar measure of accumulated plastic strain is crucial for assessing failure points^31^. As shown, FEM results closely match experimental cracks, particularly at stress concentrations near the inclined leg and perforations. This validates the CDP model’s capacity for capturing failure behavior.

#### Effect of concrete compressive strength on crack pattern and transverse compressive capacity of SEAHIVE^®^

**Fig. 7:**
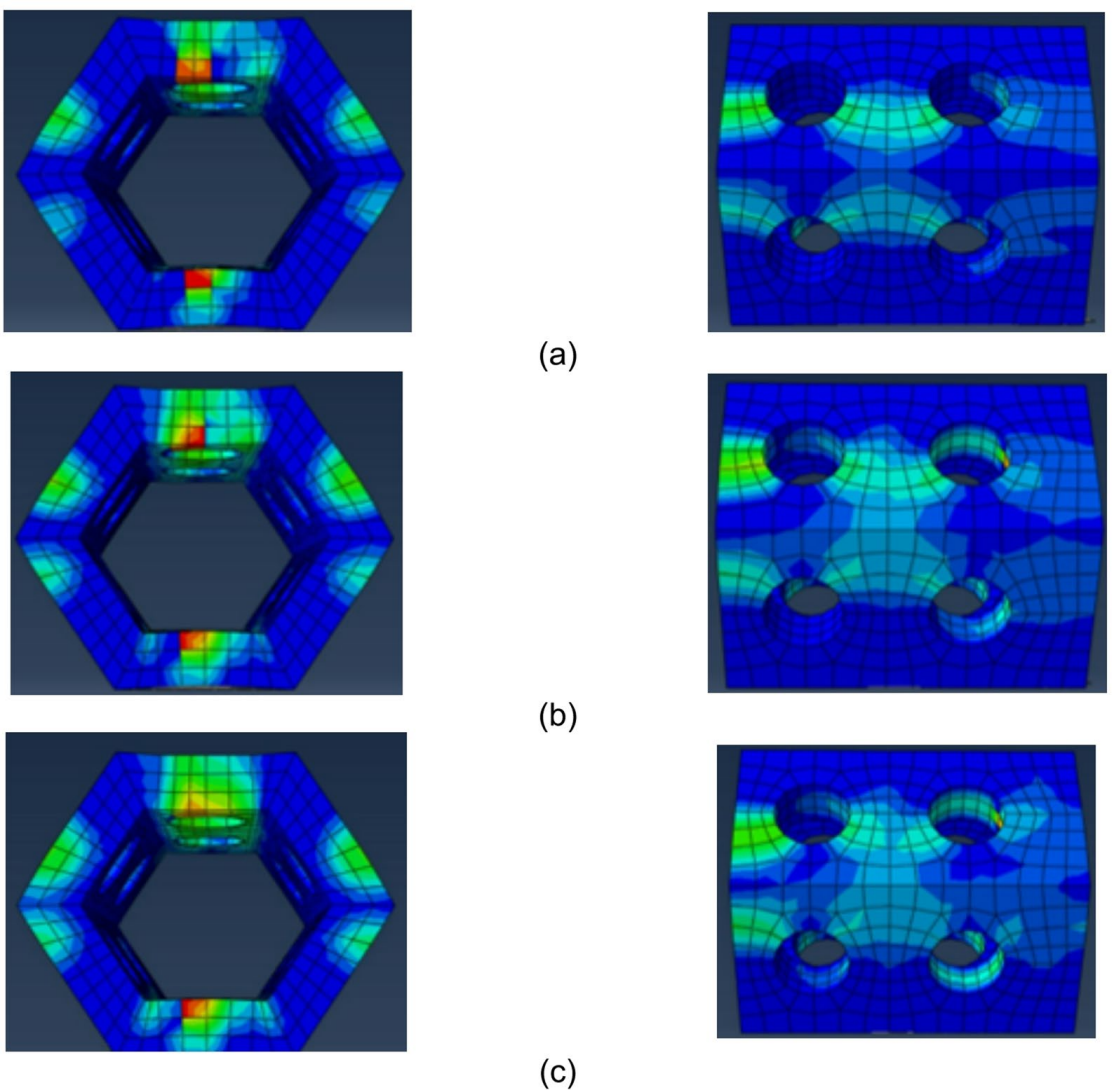
Crack propagation in elements with **a**) 40 MPa, (**b**) 30 MPa, and **c**) 20 MPa concrete compressive strength.

To expand on the baseline model developed using 20 MPa concrete, further simulations were performed with higher compressive strengths of 30 MPa and 40 MPa. These analyses aimed to assess how varying concrete strength influences the development and spread of cracks. As shown in Fig. 7, concrete with greater strength exhibited improved resistance to crack formation. Specifically, the 40 MPa model exhibited tightly confined cracking, primarily near the perforations and load-introduction points. In contrast, the 20 MPa model revealed broader cracking patterns and more extensive damage, especially in regions subjected to high stress due to perforations. The 30 MPa model exhibited behavior intermediate between the two.

These differences are largely attributed to the ability of higher-strength concrete to better resist and distribute tensile stresses, which helps to localize failure and delay extensive cracking. Perforations act as natural stress risers, and their influence becomes more pronounced in lower-strength concrete, leading to more widespread damage. Moreover, higher-strength concrete typically fails in a more brittle manner, producing sharper, more localized cracks, whereas lower-strength concrete tends to fail in a more ductile manner, with greater crack dispersion. Overall, the results underline the critical role of concrete strength in managing crack development and enhancing the structural integrity of perforated concrete elements.

Table 4 highlights the influence of concrete compressive strength on the load-bearing capacity of SEAHIVE^®^ units, keeping thickness constant. Increasing the concrete strength from 20 MPa to 40 MPa resulted in a marked improvement in transverse compressive resistance. The 40 MPa configuration achieved the highest capacity, showing a substantial enhancement over the 20 MPa case. Meanwhile, the 30 MPa element showed moderate gains, bridging the performance gap between the lower- and higher-strength cases. These results indicate that higher-strength concrete provides notable benefits in terms of structural reliability under transverse compressive loading, reinforcing its importance in the optimization of perforated concrete design.

**Table 4:** Effect of concrete compressive strength on the transverse compressive capacity of the element.

ID	Thickness (mm)	Concrete compressive strength (MPa)	Compressive capacity (kN)	Change
FCB_40MPa_D200_ T140	140	40	287.3	60%
FCB_30MPa_D200_ T140		30	213.8	19%
FCB_20MPa_D200_ T140		20	179.1	--

#### Effect of perforation’s diameter on crack pattern and transverse compressive capacity of SEAHIVE^®^

**Fig. 8:**
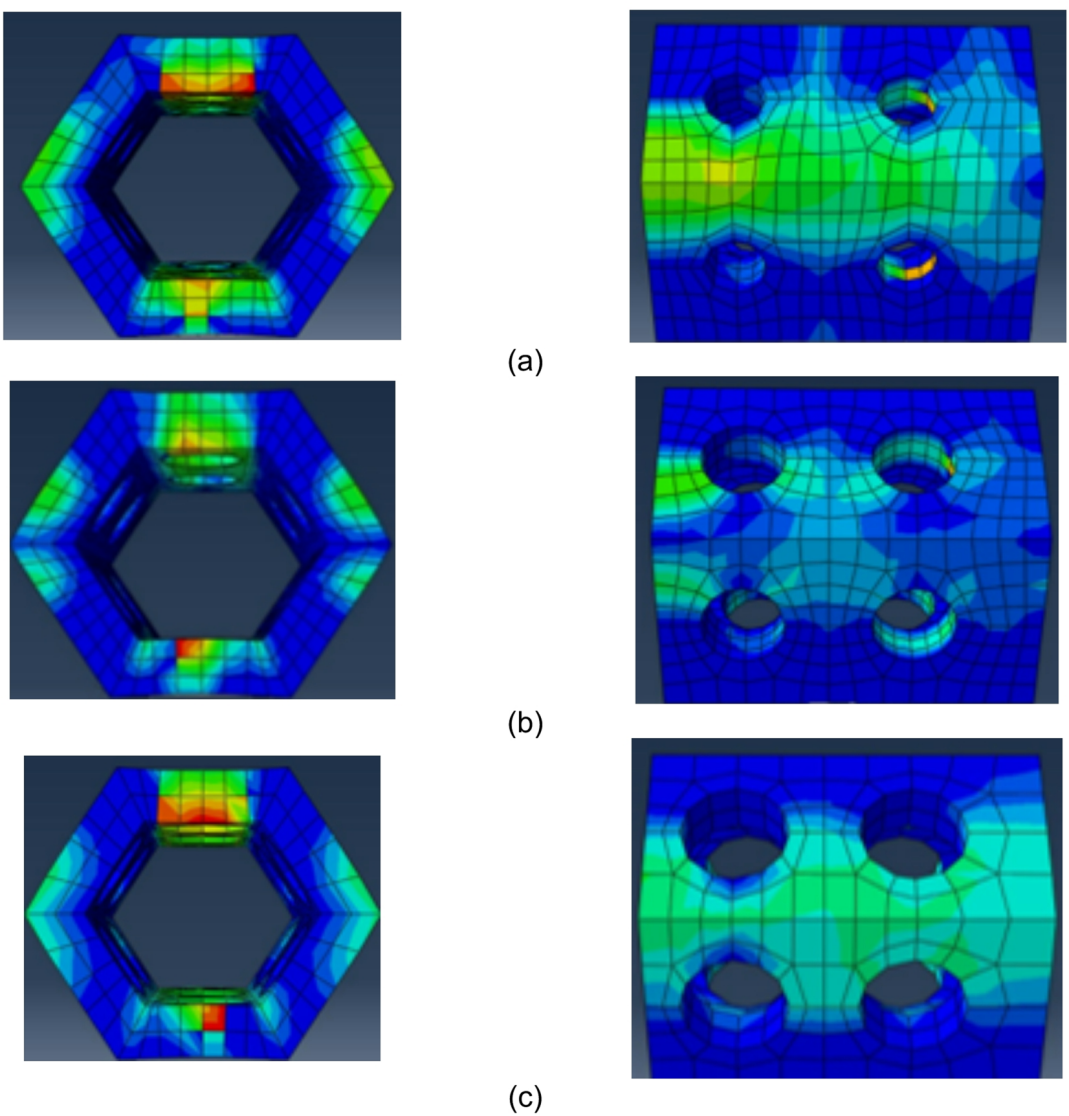
Effect of perforation diameter of SEAHIVE on crack location (**a**) 150 mm perforation diameter (**b**) 200 mm perforation diameter and (**c**) 250 mm perforation diameter.

Figure 8 illustrates how varying perforation diameters affect crack development in SEAHIVE^®^ units under transverse compressive loading. The three sizes examined—150 mm, 200 mm, and 250 mm—demonstrate a progressive increase in cracking severity with larger openings. The smallest perforation diameter resulted in controlled, limited cracking, primarily confined to the perforation zones. At 200 mm, cracking became more widespread, particularly along the connections between openings. When the diameter reached 250 mm, the unit experienced the most extensive cracking, spreading across broader regions of the cross-section. This behavior suggests that increasing perforation size intensifies stress concentrations, reducing the element’s ability to sustain compressive loads and underscoring the importance of careful sizing in design.

Table 5 presents the transverse compressive capacities of SEAHIVE^®^ units across various concrete strengths and perforation diameters. Across all strength levels (20 MPa, 30 MPa, and 40 MPa), increasing the perforation size from 150 mm to 250 mm consistently reduced structural performance. At 20 MPa, for example, the unit with 150 mm perforations performed better than those with larger openings. Similar reductions in capacity were observed for 30 MPa and 40 MPa concrete, reinforcing the pattern. These findings suggest that enlarging perforation diameters compromises structural efficiency by diminishing the load-carrying area, making the units more susceptible to localized failures under compressive loading.

**Table 5:** Effect of different perforation diameters on transverse compressive strength of the element with different compressive strength of concrete.

ID	Thickness (mm) ($\:\boldsymbol{T}$)	Concrete compressive strength (MPa) ( $\:{\boldsymbol{f}}_{\boldsymbol{c}}^{\boldsymbol{{\prime\:}}}$)	Perforation diameter (mm) ($\:{\boldsymbol{P}}_{\boldsymbol{d}}$)	Compressive capacity (kN) ($\:\boldsymbol{C}\boldsymbol{c}\boldsymbol{a}\boldsymbol{p})$
FCB_20MPa_D150_T140	140	20	150	200.2
FCB_20MPa_D200_T140			200	179.1
FCB_20MPa_D250_T140			250	226.2
FCB_30MPa_D150_T140	140	30	150	241.3
FCB_30MPa_D200_T140			200	213.9
FCB_30MPa_D250_T140			250	235.0
FCB_40MPa_D150_T140	140	40	150	344.9
FCB_40MPa_D200_T140			200	287.3
FCB_40MPa_D250_T140			250	306.4

#### Effect of thickness on crack pattern and transverse compressive capacity of SEAHIVE^®^

**Fig. 9:**
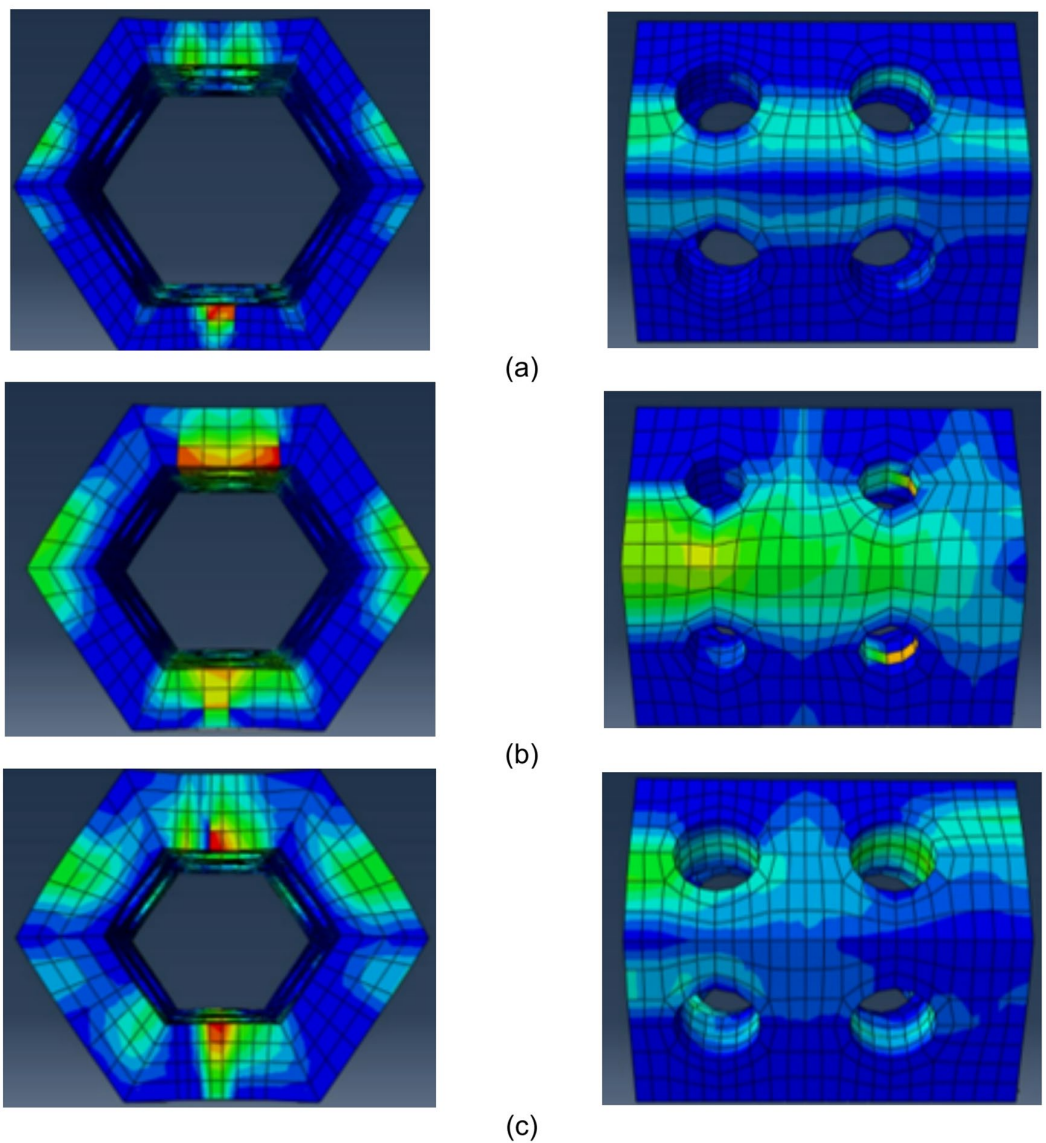
Effect thickness of SEAHIVE on crack location (**a**) 100 mm (**b**) 140 mm, and (**c**) 180 mm thicknesses.

Figure 9 illustrates the crack propagation locations in elements with a compressive strength of 20 MPa and thickness of 100 mm, 140 mm, and 180 mm, respectively. The analysis demonstrates that increasing the thickness of concrete elements with a compressive strength of 20 MPa significantly reduces crack propagation and enhances structural integrity. The thinnest element (100 mm) exhibits extensive crack propagation, with cracks spreading across the cross-section due to insufficient material to effectively resist stress concentrations around the perforations. In contrast, the 140 mm element shows improved performance, with cracks becoming more localized around the perforations, indicating a better balance between material usage and stress redistribution. The thickest element (180 mm) performs the best, displaying minimal crack propagation and highly localized cracking near the perforation zones, thanks to the additional material that resists the applied stresses. This behavior highlights the critical role of thickness in structural performance: thinner elements are more vulnerable to failure due to their inability to manage stress concentrations. In comparison, thicker elements distribute stresses more effectively, ensuring greater durability and load-bearing capacity. Optimizing thickness is therefore crucial for designing efficient and reliable structural elements, especially when perforations or stress-concentrating features are present.

Table 6 illustrates the significant influence of element thickness on the transverse compressive capacity of concrete with a fixed perforation diameter of 200 mm across various compressive strengths. Increasing thickness consistently enhances the transverse compressive capacity, as observed across all strength levels (20 MPa, 30 MPa, and 40 MPa). For instance, at 20 MPa, the compressive capacity increased from 79.5 kN at 100 mm thickness to 179.1 kN at 140 mm and 303.0 kN at 180 mm. Similarly, at 40 MPa, the capacity rose from 122.3 kN at 100 mm thickness to 287.3 kN at 140 mm and reached 470.2 kN at 180 mm. This trend can be attributed to the increase in the load-bearing cross-sectional area with greater thickness, which enhances the element’s ability to resist transverse compressive loads.

**Table 6:** Effect of different thickness on transverse compressive strength of the element with different compressive strength of concrete.

ID	Thickness (mm) ($\:\boldsymbol{T}$)	Concrete compressive strength (MPa) ( $\:{\boldsymbol{f}}_{\boldsymbol{c}}^{\boldsymbol{{\prime\:}}}$)	Perforation diameter (mm) ($\:{\boldsymbol{P}}_{\boldsymbol{d}}$)	Compressive capacity (kN) ($\:\boldsymbol{C}\boldsymbol{c}\boldsymbol{a}\boldsymbol{p})$
FCB_20MPa_D200_T100	100	20	200	79.5
FCB_20MPa_D200_T140	140		200	179.1
FCB_20MPa_D200_T180	180		200	303.0
FCB_30MPa_D200_T100	100	30	200	93.3
FCB_30MPa_D200_T140	140		200	213.9
FCB_30MPa_D200_T180	180		200	346.1
FCB_40MPa_D200_T100	100	40	200	122.3
FCB_40MPa_D200_T140	140		200	287.3
FCB_40MPa_D200_T180	180		200	470.2

Table 7 illustrates the relationships among concrete compressive strength, perforation diameter, and element thickness, and their impact on transverse compressive capacity. The data shows that increasing thickness and compressive strength consistently improve transverse compressive capacity, as thicker and stronger materials better resist applied loads. For instance, with a 200 mm perforation, the transverse compressive capacity increases from 79.5 kN for a 100 mm-thick element to 303.0 kN for a 180 mm-thick element, assuming a compressive strength of 20 MPa. Similarly, higher compressive strength leads to better performance across all thicknesses and perforation diameters; for a 140 mm-thick element with a 150 mm perforation, the transverse compressive capacity increases from 200.2 kN (20 MPa) to 344.9 kN (40 MPa). Larger perforations, however, reduce capacity due to increased stress concentrations, whereas smaller perforations yield better structural performance.

**Table 7:** The transverse compressive capacity of SEAHIVE^®^ elements with different concrete compressive strengths and perforation diameters.

ID	Thickness (mm) ($\:\boldsymbol{T}$)	Concrete compressive strength (MPa) ( $\:{\boldsymbol{f}}_{\boldsymbol{c}}^{\boldsymbol{{\prime\:}}}$)	Perforation diameter (mm) ($\:{\boldsymbol{P}}_{\boldsymbol{d}}$)	Compressive capacity (kN) ($\:\boldsymbol{C}\boldsymbol{c}\boldsymbol{a}\boldsymbol{p})$
FCB_20MPa_D150_T100	100	20	150	75.31
FCB_20MPa_D200_T100			200	79.5
FCB_20MPa_D250_T100			250	70.97
FCB_20MPa_D150_T140	140		150	200.2
FCB_20MPa_D200_T140			200	179.1
FCB_20MPa_D250_T140			250	226.2
FCB_20MPa_D150_T180	180		150	326.9
FCB_20MPa_D200_T180			200	303.0
FCB_20MPa_D250_T180			250	200.3
FCB_30MPa_D150_T100	100	30	150	90.0
FCB_30MPa_D200_T100			200	93.3
FCB_30MPa_D250_T100			250	79.8
FCB_30MPa_D150_T140	140		150	241.3
FCB_30MPa_D200_T140			200	213.9
FCB_30MPa_D250_T140			250	235.0
FCB_30MPa_D150_T180	180		150	371.2
FCB_30MPa_D200_T180			200	346.1
FCB_30MPa_D250_T180			250	240.4
FCB_40MPa_D150_T100	100	40	150	116.3
FCB_40MPa_D200_T100			200	122.3
FCB_40MPa_D250_T100			250	98.2
FCB_40MPa_D150_T140	140		150	344.9
FCB_40MPa_D200_T140			200	287.3
FCB_40MPa_D250_T140			250	306.4
FCB_40MPa_D150_T180	180		150	520.1
FCB_40MPa_D200_T180			200	470.2
FCB_40MPa_D250_T180			250	341.6

A comparative analysis of parameter sensitivity indicates that thickness is the dominant factor governing transverse compressive capacity, producing increases exceeding 280% across the studied range. In contrast, concrete compressive strength improved by up to 60%, while perforation diameter exhibited a secondary but notable influence due to stress-concentration effects.

### Specimens under flexure

Holes on all surfaces of the hexagonal, hollow, perforated specimen complicate the analysis. Moreover, the shear span-to-reinforcement depth ratio of the SEAHIVE^®^ was equal to 0.8; therefore, conventional beam theory is not appropriate for its analysis. Because of the mentioned issue, the FEM was employed to evaluate the element’s performance under flexure. The purpose of developing the FEM model was to simulate crack propagation with special attention to the inclined leg. Material properties were defined based on experimental data, and the concrete damage plasticity (CDP) model was used to represent concrete with a compressive strength of 20 MPa. A static general procedure was adopted for the analysis.

**Fig. 10:**
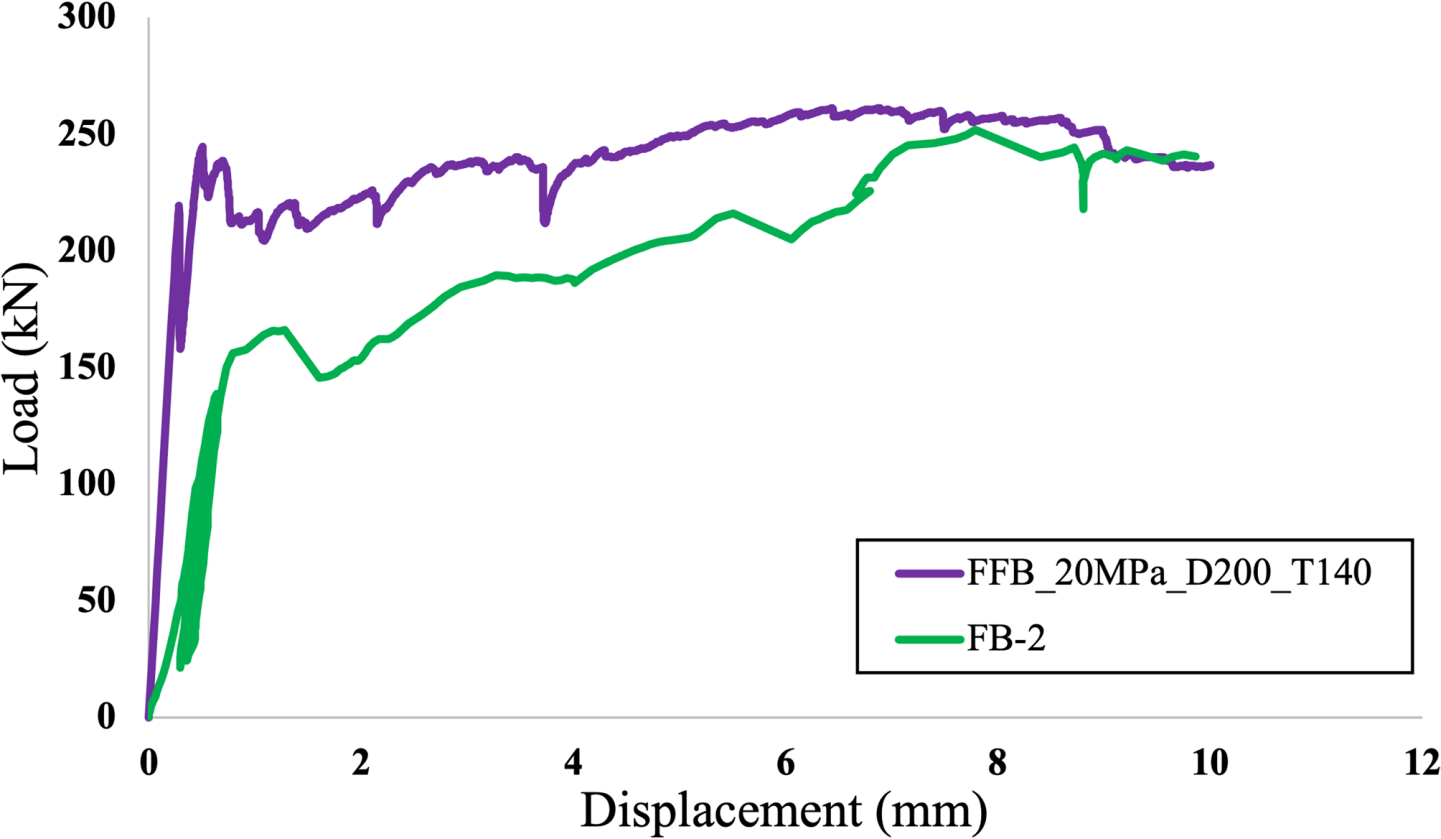
The load-displacement curve obtained from FEM and Experiments.

Furthermore, displacement was applied rather than the load directly to the top of the sample. This approach helps minimize potential errors during the analysis. Figure 10 shows the load-displacement curves obtained from FEM and experiments. The numerical model exhibited a higher load level up to a deflection of approximately 7.7 mm, likely due to imperfections present during loading in experiments. Beyond this Deflection, the load predicted by the analytical model aligns with the experimental results.

**Fig. 11:**
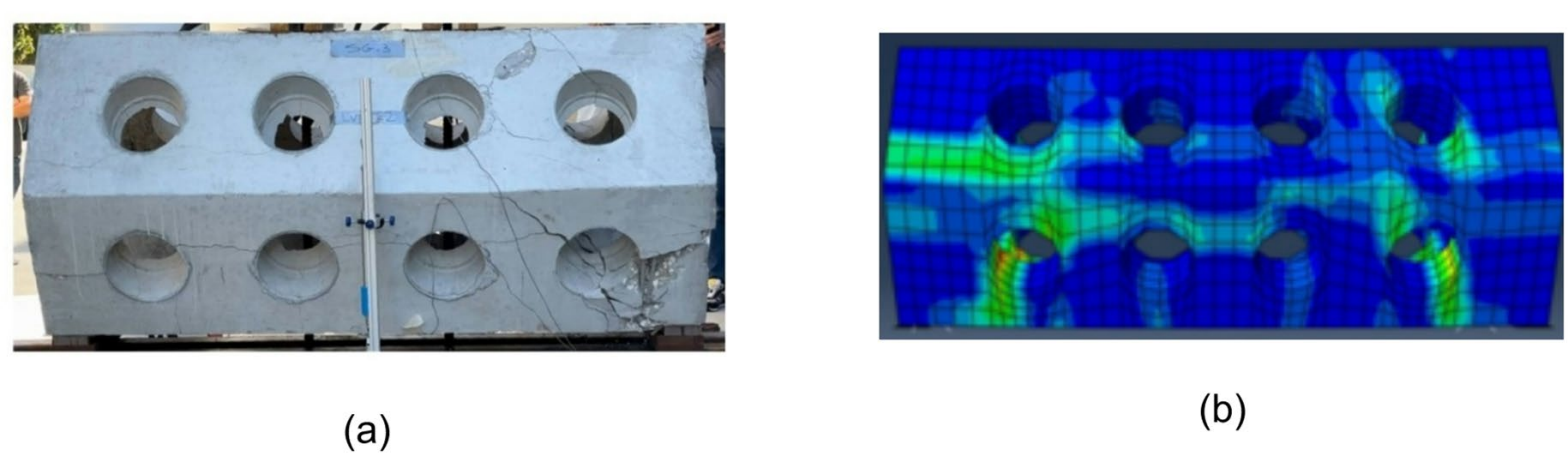
Comparing the crack propagation in (**a**) experiment (**b**) FEM.

Figure 11 shows the PEMAG contour, which represents the plastic strain magnitude, and compares crack propagation in experiments with that in the FEM model. As shown in the figure, the FEM model accurately predicts the locations and patterns of crack propagation observed experimentally, particularly around stress-concentration areas near the perforations, thereby validating the model’s ability to simulate structural behavior under loading conditions.

#### Effect of concrete compressive strength on crack pattern and flexural capacity of SEAHIVE^®^

**Fig. 12:**
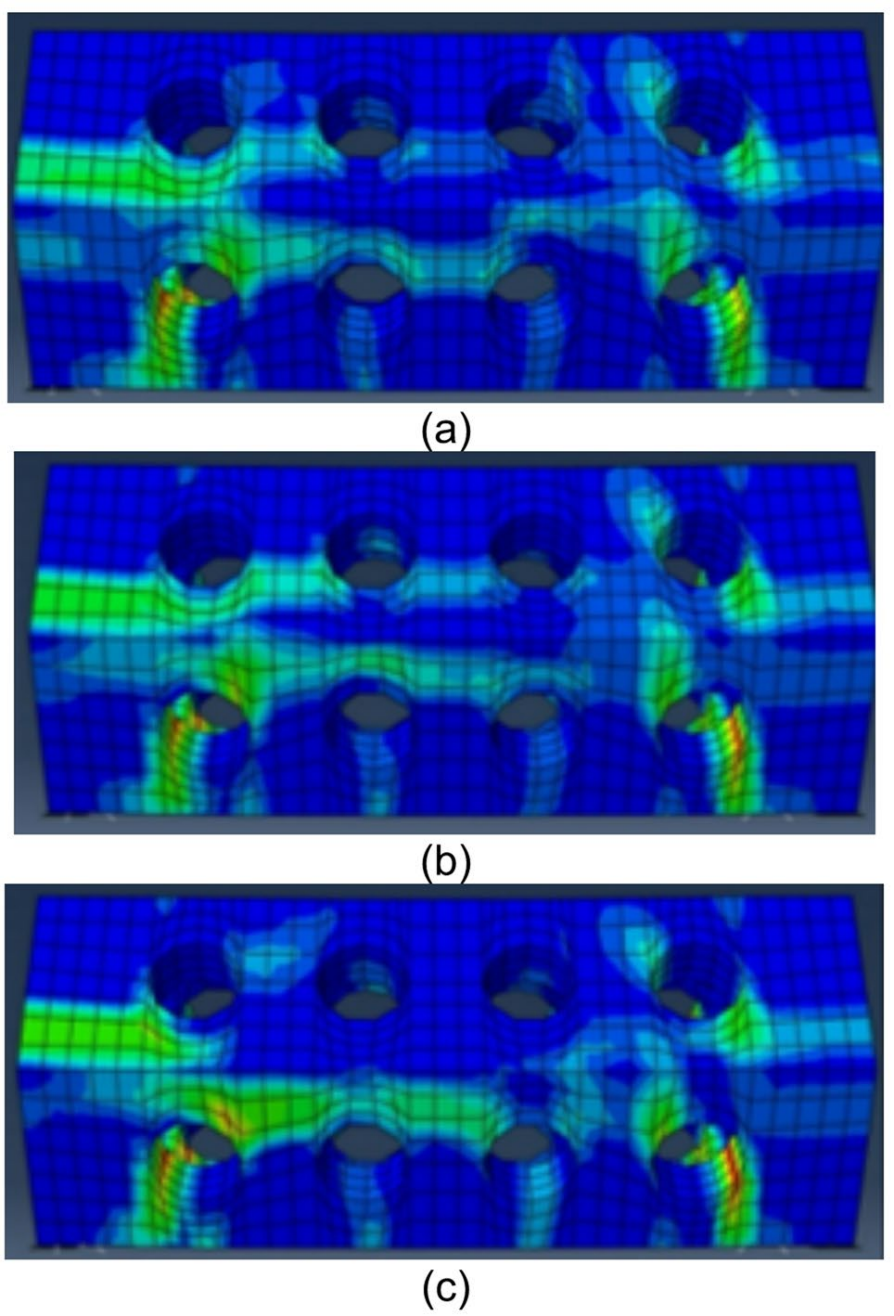
Crack propagation in elements with **a**) 20 MPa, (**b**) 30 MPa, and **c**) 40 MPa concrete compressive strength.

Following the initial modeling of the element using a 20 MPa CDP model, additional models were created with concrete strengths of 40 MPa and 30 MPa to observe and analyze crack propagation within the element. Figure 12 illustrates the effect of concrete compressive strength (20 MPa, 30 MPa, and 40 MPa) on crack propagation in perforated beam elements under four-point bending as simulated in the FEM model. The element with 20 MPa concrete shows the most extensive crack propagation, particularly around the perforations, due to its lower compressive strength and reduced capacity to withstand tensile stresses. The element with 30 MPa concrete exhibits moderate crack propagation, reflecting improved crack resistance as concrete strength increases. The element with 40 MPa concrete shows minimal crack propagation, demonstrating the superior performance of higher-strength concrete in resisting stress concentrations and mitigating failure. This analysis highlights the significant role of compressive strength in enhancing the structural integrity of perforated beams under flexural loading.

Table 8 summarizes the effect of concrete compressive strength on the flexural capacity of perforated beam elements. As the compressive strength increases from 20 MPa to 40 MPa, the element’s flexural capacity improves significantly. The element with 20 MPa concrete has a flexural capacity of 261.3 kN, increasing to 347.9 kN (a 32% improvement) with 30 MPa concrete and to 434.8 kN (a 66% improvement) with 40 MPa concrete. This trend clearly demonstrates that higher concrete compressive strength enhances the element’s flexural performance.

**Table 8:** Effect of concrete compressive strength on the compressive capacity of the element.

ID	Thickness (mm) ($\:\boldsymbol{T}$)	Concrete compressive strength (MPa) ( $\:{\boldsymbol{f}}_{\boldsymbol{c}}^{\boldsymbol{{\prime\:}}}$)	Perforation diameter (mm) ($\:{\boldsymbol{P}}_{\boldsymbol{d}}$)	Change
FFB_40MPa_D200_ T140	140	40	434.8	66%
FFB_30MPa_ D200_ T140		30	347.9	32%
FFB_20MPa_ D200_ T140		20	261.3	--

#### Effect of perforation’s diameter on crack pattern and flexural capacity of SEAHIVE^®^

**Fig. 13:**
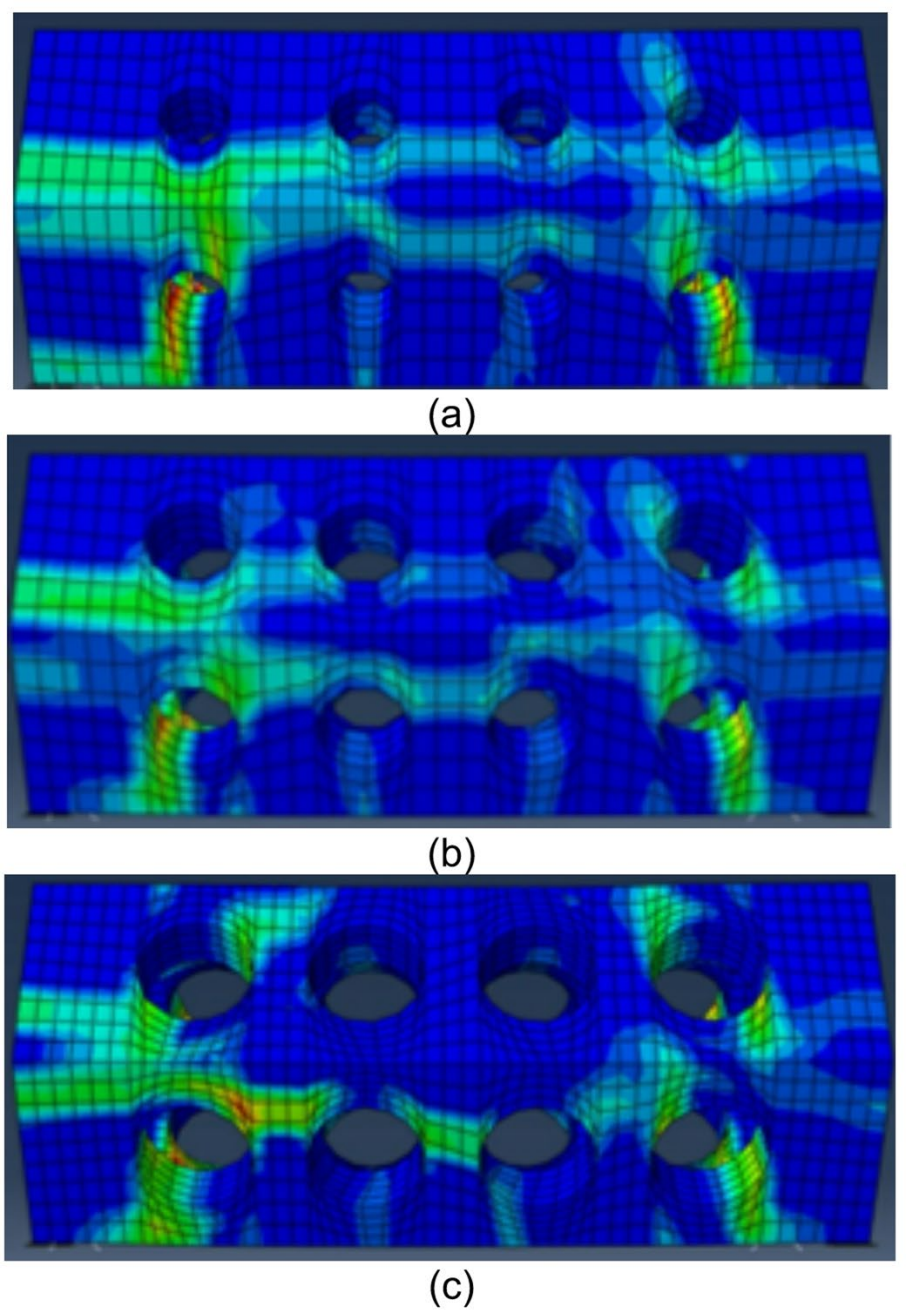
Effect of perforation diameter of SEAHIVE on crack location (**a**) 150 mm perforation diameter (**b**)200 mm perforation diameter and (**c**) 250 mm perforation diameter.

Figure 13 examines the effect of perforation diameter (150 mm, 200 mm, and 250 mm) on crack propagation in elements with a concrete compressive strength of 20 MPa. Crack patterns reveal that as the perforation diameter increases, stress concentration around the perforations becomes more pronounced, leading to more extensive crack propagation. Crack propagation is relatively limited for the element with a 150 mm perforation diameter due to smaller stress concentrations in the 200 mm perforation diameter; crack propagation increases, particularly around the perforation edges. The 250 mm-diameter perforation element exhibits the most extensive crack development, indicating that larger perforations significantly weaken the element by amplifying stress-concentration zones. This analysis highlights that increasing perforation size adversely affects the structural integrity of elements.

Table 9 indicates that the perforation diameter significantly affects the compressive capacity of fully filled concrete elements, with larger diameters reducing flexural capacity across all tested compressive strengths (20 MPa, 30 MPa, and 40 MPa). For instance, at 20 MPa, the compressive capacity decreased from 280.3 kN for a 150 mm perforation diameter to 261.3 kN for 200 mm and further to 206.1 kN for 250 mm. Similar trends were observed at higher compressive strengths, with the capacity decreasing from 488.0 kN to 434.8 kN and 366.6 kN at 40 MPa as the perforation diameter increased from 150 mm to 200 mm and 250 mm, respectively. This reduction is due to the reduced effective cross-sectional area available to resist flexural loads as the perforation size increases.

**Table 9:** Effect of different perforation diameters on the flexural capacity of the element with different compressive strengths of concrete.

ID	Thickness (mm) ($\:\boldsymbol{T}$)	Concrete compressive strength (MPa) ( $\:{\boldsymbol{f}}_{\boldsymbol{c}}^{\boldsymbol{{\prime\:}}}$)	Perforation diameter (mm) ($\:{\boldsymbol{P}}_{\boldsymbol{d}}$)	Flexural capacity (kN) $\:\boldsymbol{F}\boldsymbol{c}\boldsymbol{a}\boldsymbol{p})$
FFB_20MPa_D150_T140	140	20	150	280.3
FFB_20MPa_D200_T140			200	261.3
FFB_20MPa_D250_T140			250	206.1
FFB_30MPa_D150_T140	140	30	150	387.9
FFB_30MPa_D200_T140			200	347.9
FFB_30MPa_D250_T140			250	269.6
FFB_40MPa_D150_T140	140	40	150	488.0
FFB_40MPa_D200_T140			200	434.8
FFB_40MPa_D250_T140			250	366.6

#### Effect of thickness o on crack pattern and flexural capacity of SEAHIVE^®^

**Fig. 14:**
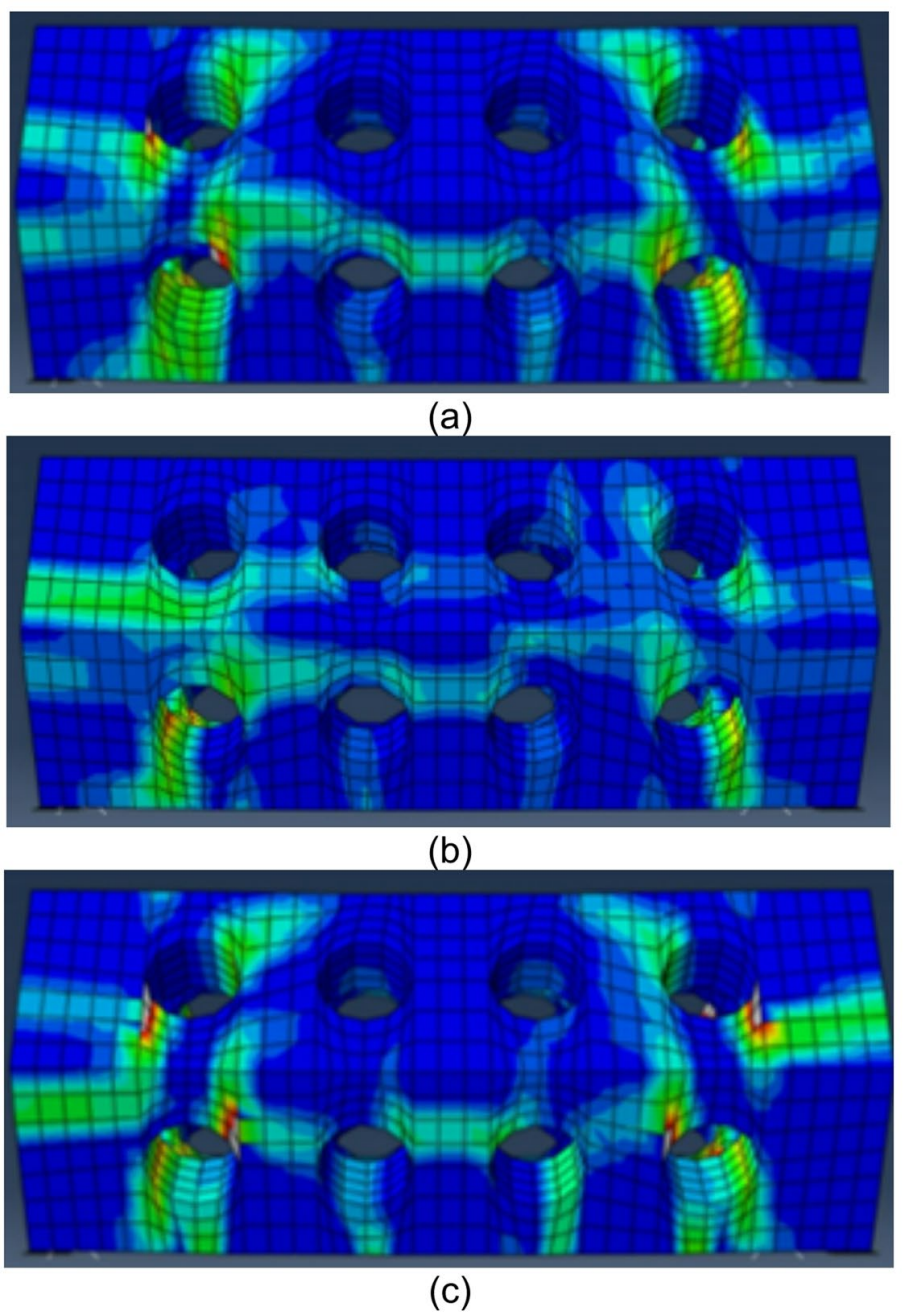
Effect thickness of SEAHIVE under flexural loads on crack location (**a**) 100 mm (**b**) 140 mm, and (**c**) 180 mm thicknesses.

Figure 14 illustrates the effect of thickness (100 mm, 140 mm, and 180 mm) on crack propagation in perforated elements with a concrete compressive strength of 20 MPa under flexural loading. As the thickness increases, the extent of crack propagation decreases significantly, with the 100 mm-thick elements exhibiting the most severe cracking, particularly around the perforation edges. The 140 mm-thick element shows moderate crack propagation, while the 180 mm-thick element demonstrates minimal cracking, indicating improved structural integrity with increased thickness. These results highlight that increasing the element thickness enhances its resistance to stress concentrations and mitigates crack formation under flexural loads.

Table 10 demonstrates the critical role of panel thickness in enhancing the flexural capacity of fully filled concrete elements with a fixed perforation diameter of 200 mm. Across all tested compressive strengths (20 MPa, 30 MPa, and 40 MPa), an increase in thickness led to a substantial rise in flexural capacity. For instance, at a compressive strength of 20 MPa, the flexural capacity increased from 137.8 kN at 100 mm thickness to 261.3 kN at 140 mm and 339.6 kN at 180 mm. Similarly, at 40 MPa, the flexural capacity rose from 339.6 kN at 100 mm thickness to 434.8 kN at 140 mm, peaking at 581.7 kN at 180 mm. This trend is attributed to the greater moment of inertia provided by thicker panels, which improves their resistance to bending and deformation under load. These findings underscore that panel thickness is a key parameter for optimizing flexural performance in structural elements, particularly in designs requiring enhanced resistance to bending stresses.

**Table 10:** Effect of different thicknesses on the flexural strength of the element with different compressive strengths of concrete.

ID	Thickness (mm) ($\:\boldsymbol{T}$)	Concrete compressive strength (MPa) ( $\:{\boldsymbol{f}}_{\boldsymbol{c}}^{\boldsymbol{{\prime\:}}}$)	Perforation diameter (mm) ($\:{\boldsymbol{P}}_{\boldsymbol{d}}$)	Flexural capacity (kN) $\:\boldsymbol{F}\boldsymbol{c}\boldsymbol{a}\boldsymbol{p})$
FFB_20MPa_D200_T100	100	20	200	137.8
FFB_20MPa_D200_T140	140		200	261.3
FFB_20MPa_D200_T180	180		200	339.6
FFB_30MPa_D200_T100	100	30	200	198.9
FFB_30MPa_D200_T140	140		200	347.9
FFB_30MPa_D200_T180	180		200	470.4
FFB_40MPa_D200_T100	100	40	200	339.6
FFB_40MPa_D200_T140	140		200	434.8
FFB_40MPa_D200_T180	180		200	581.7

Table 11 summarizes the flexural capacity of SEAHIVE^®^ elements with varying concrete compressive strengths, perforation diameters, and thicknesses. The results indicate that increasing the concrete compressive strength significantly enhances the flexural capacity, with elements made of 40 MPa concrete consistently outperforming those with 20 MPa and 30 MPa. Similarly, increasing the element thickness from 100 mm to 180 mm improves the flexural performance, as thicker elements exhibit higher load capacities. Conversely, larger perforation diameters (e.g., 250 mm) reduce the flexural capacity due to increased stress concentrations and weakened cross-sections. These findings underscore the importance of optimizing concrete strength, thickness, and perforation size to achieve higher structural performance under flexural loads.

**Table 11:** Flexural capacity of SEAHIVE^®^ elements with different concrete flexural strengths and perforation diameters.

ID	Thickness (mm) ($\:\boldsymbol{T}$)	Concrete compressive strength (MPa) ( $\:{\boldsymbol{f}}_{\boldsymbol{c}}^{\boldsymbol{{\prime\:}}}$)	Perforation diameter (mm) ($\:{\boldsymbol{P}}_{\boldsymbol{d}}$)	Flexural capacity (kN) ($\:\boldsymbol{F}\boldsymbol{c}\boldsymbol{a}\boldsymbol{p})$
FFB_20MPa_D150_T100	100	20	150	159.4
FFB_20MPa_D200_T100			200	137.8
FFB_20MPa_D250_T100			250	113.8
FFB_20MPa_D150_T140	140		150	280.3
FFB_20MPa_D200_T140			200	261.3
FFB_20MPa_D250_T140			250	206.1
FFB_20MPa_D150_T180	180		150	406.9
FFB_20MPa_D200_T180			200	339.6
FFB_20MPa_D250_T180			250	251.9
FFB_30MPa_D150_T100	100	30	150	237.5
FFB_30MPa_D200_T100			200	198.9
FFB_30MPa_D250_T100			250	160.9
FFB_30MPa_D150_T140	140		150	387.9
FFB_30MPa_D200_T140			200	347.9
FFB_30MPa_D250_T140			250	269.6
FFB_30MPa_D150_T180	180		150	535.2
FFB_30MPa_D200_T180			200	470.4
FFB_30MPa_D250_T180			250	333.8
FFB_40MPa_D150_T100	100	40	150	373.8
FFB_40MPa_D200_T100			200	339.6
FFB_40MPa_D250_T100			250	208.4
FFB_40MPa_D150_T140	140		150	488.0
FFB_40MPa_D200_T140			200	434.8
FFB_40MPa_D250_T140			250	366.6
FFB_40MPa_D150_T180	180		150	670.6
FFB_40MPa_D200_T180			200	581.7
FFB_40MPa_D250_T180			250	432.1

Under flexural loading, thickness and concrete compressive strength exhibited comparable influence on capacity enhancement, whereas perforation diameter consistently reduced capacity due to a reduction in effective section modulus. These findings suggest that thickness primarily governs compression-dominated behavior, while both thickness and concrete strength play significant roles in flexure-dominated response.

The database used in this study comprises 27 flexural and 27 compression data points generated from validated finite element simulations of SEAHIVE^®^ structural configurations under varying geometric and concrete compressive strength. Prior to developing the predictive model, the dataset was examined statistically to evaluate the distribution and variability of the input parameters.

Descriptive statistical indicators, including minimum, maximum, mean, and standard deviation, were calculated for all parameters in order to understand the range and dispersion of the variables used in the modeling process. These statistical measures confirm that the selected parameters cover a meaningful range of structural configurations relevant to the SEAHIVE^®^ system.

Table 12 summarizes the statistical characteristics of the key parameters used in the compression and flexural datasets. The results show consistent distributions for thickness, compressive strength, and perforation diameter across both datasets, with identical standard deviations and similar ranges, indicating uniform input conditions. In the compression data, the average compressive capacity is 228.88 kN, with a relatively wide range, whereas in the flexural data, the average flexural capacity is 333.14 kN, also exhibiting significant variability. These variations in capacity values suggest a strong sensitivity of structural performance to the governing parameters, while the consistency in input statistics ensures a reliable basis for comparative analysis.

**Table 12:** Statistical analysis of the parameters.

Test	Statistic Metric	Thickness (mm) ($\:\boldsymbol{T}$)	Concrete compressive strength (MPa) ( $\:{\boldsymbol{f}}_{\boldsymbol{c}}^{\boldsymbol{{\prime\:}}}$)	Perforation diameter (mm) ($\:{\boldsymbol{P}}_{\boldsymbol{d}}$)	Flexural capacity (kN) ($\:\boldsymbol{F}\boldsymbol{c}\boldsymbol{a}\boldsymbol{p})$	Compressive capacity (kN) ($\:\boldsymbol{C}\boldsymbol{c}\boldsymbol{a}\boldsymbol{p})$
Compression data	Standard Deviation	34.64	10.00	41.60	-	124.69
	Average	140.00	30.00	200.00	-	228.88
	Maximum	100.00	20.00	150.00	-	70.97
	Minimum	180.00	40.00	250.00	-	520.10
Flexural data	Standard Deviation	34.64	10.00	41.60	140.68	-
	Average	140.00	30.00	200.00	333.14	-
	Maximum	100.00	20.00	150.00	113.80	-
	Minimum	180.00	40.00	250.00	670.60	-

## Differential evolutionary chromosomal gene expression programming

To complement FEM analysis and provide engineers with accessible predictive tools, this study also incorporates an artificial intelligence-based approach to model the complex behavior of SEAHIVE^®^ units. Given the challenges in deriving accurate analytical expressions for structural systems with irregular geometries and perforations, evolutionary algorithms offer an efficient alternative for developing symbolic regression models that generate explicit mathematical expressions for predicting both transverse compressive and flexural capacities. This section describes the Differential Evolutionary Chromosomal Gene Expression Programming (DEC-GEP)^32^ technique employed in this study and explains how it was used to derive practical design equations aligned with the numerical analysis results.

**Fig. 15:**
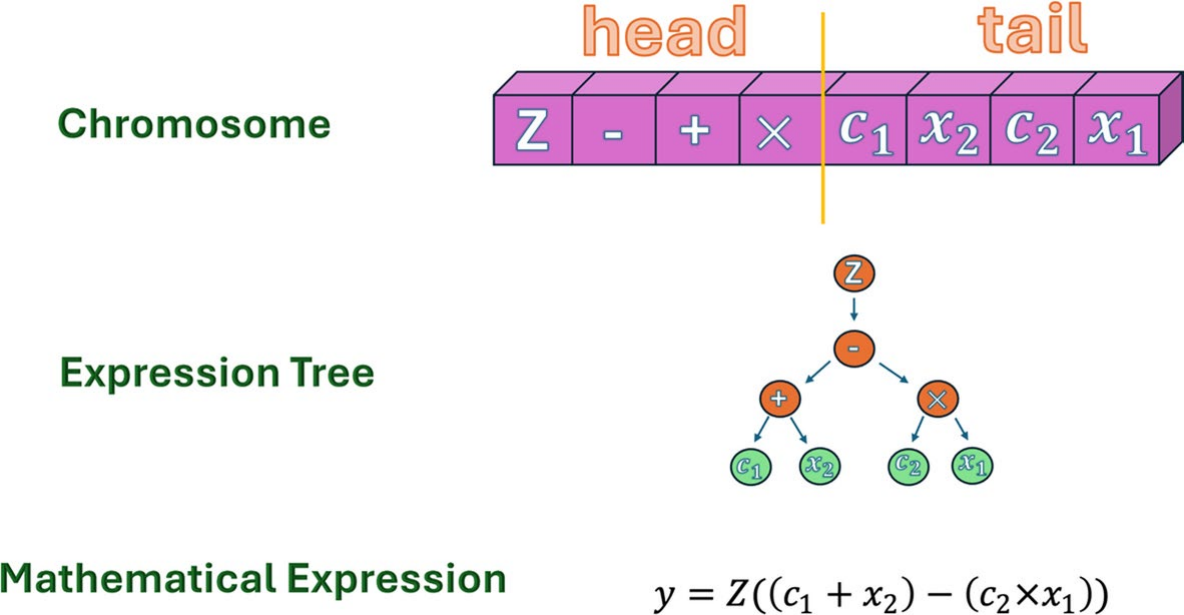
A classical GEP Program.

The fixed chromosome structure of Gene Expression Programming (GEP)^33^ contributes to its popularity as an evolutionary algorithm since it enables more dependable and efficient creation of expression trees than other Genetic Programming (GP) methods^34,35^. Figure 15 shows an example of a GEP chromosome, together with its corresponding expression tree (ET) and the mathematical expression it produces, which serves as a program. GEP-based Symbolic Regression (SR)^35,36^ models can identify the relationship patterns between inputs and outputs within datasets.

The latest GEP versions enable solutions comprising multiple expression trees, which researchers call multi-gene models^37^. The GEP chromosome consists of symbols arranged in a fixed-length array, containing operators, variables, and constants. The decoding of these arrays is performed using Karva notation (K-expression)^33^, which yields valid expression trees and mathematical formulas. A typical GEP chromosome has two segments: (a) the head section contains symbols that enable the generation and expansion of expression trees; and (b) the GEP chromosome’s tail section contains only symbols representing variables and constants. The tail segment ensures proper expression tree completion, producing valid mathematical models. The structured configuration enables accurate and flexible development of predictive models through GEP.

The Differential Evolution (DE)^38^ algorithm stands out as an effective population-based optimization tool recognized for its straightforward approach to solving complex nonlinear multi-dimensional problems. Storn and Price^38^ developed DE to improve candidate solutions through iterative processes that apply mutation, crossover, and selection strategies based on differential comparisons between randomly chosen population members. Unlike gradient-based methods, the DE algorithm stands out because it operates without derivative information, which makes it ideal for black-box optimization problems. Successful applications of DE in structural optimization, machine learning, and symbolic regression emerged because of its skill to maintain population diversity while balancing exploration and exploitation.

Several studies have employed the DE algorithm to optimize expression tree genotypes and constant terminals within GEP frameworks^34,39,40^. In^32^, integrating a head root and a numerical box section into the GEP chromosome was proposed to enhance expression efficiency. Including the head root eliminates redundant coding at the beginning of the chromosome, thereby improving the overall coding efficiency. Additionally, the numerical box section, positioned after the tail part of the chromosome, stores the numerical values of constant terminals used in constructing expression trees. This configuration supports the simultaneous evolution of constant values alongside other expression tree elements. By expanding the chromosome design, the study effectively completes the search space for expression trees, facilitating more effective optimization and the discovery of globally optimal solutions.

The root node, derived from the function set ($\:F$), initiates the expression tree at the beginning of the head section. Constructing a GEP chromosome requires defining a function set $\:F=\{{f}_{1},\:{f}_{2},{f}_{3},\dots\:,{f}_{n}\}$, a variable set $\:V=\{{x}_{1},\:{x}_{2},{x}_{3},\dots\:,{x}_{m}\}$, and a constant set $\:C=\{{c}_{1},\:{c}_{2},{c}_{3},\dots\:,{c}_{k}\}$. The function set includes operators and mathematical functions used in the expression tree, the variable set defines the input terminals, and the constant set contains fixed numerical parameters. In this study, a configuration with $\:F=\{\times\:,\:\div,+,-,\sqrt{\:}\}$, $\:V=\{T,\:\:{f}_{c}^{{\prime\:}},{P}_{d}\}$, and $\:C=0$ enables the formation of five mathematical expressions comprising four variables and zero constants, using functions from the specified set.

**Fig. 16:**
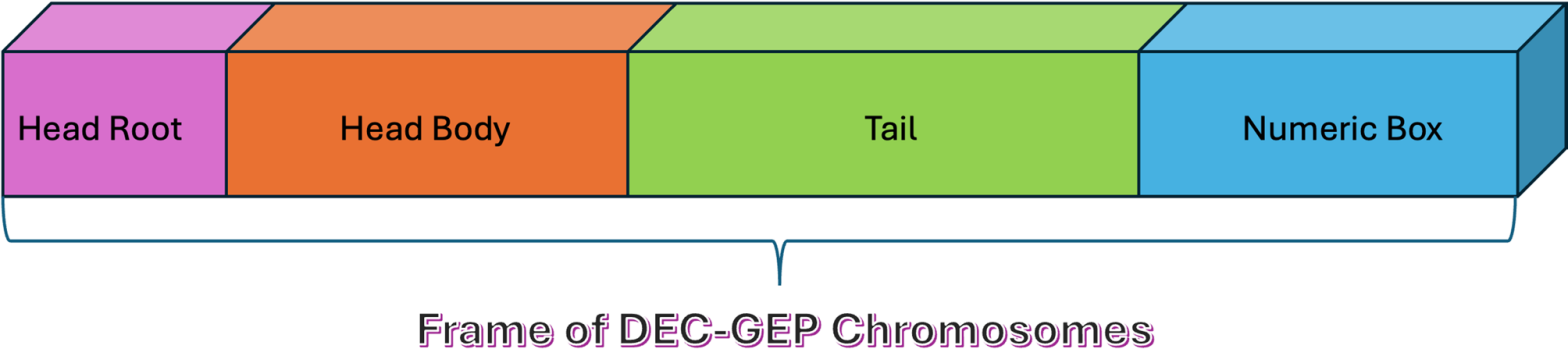
DEC-GEP frame structure.

The chromosome frame structure and code allocation determine optimization efficiency for GEP chromosomes. As illustrated in Fig. 16, DEC-GEP chromosomes start with a start codon that starts the creation of a valid expression tree and function. This feature optimizes coding efficiency by removing unnecessary codes before the expression tree starts in the head section. The DE algorithm becomes more effective in chromosome optimization because the improved code structure addresses the influence of problem dimensionality on metaheuristic algorithm performance.

**Fig. 17:**
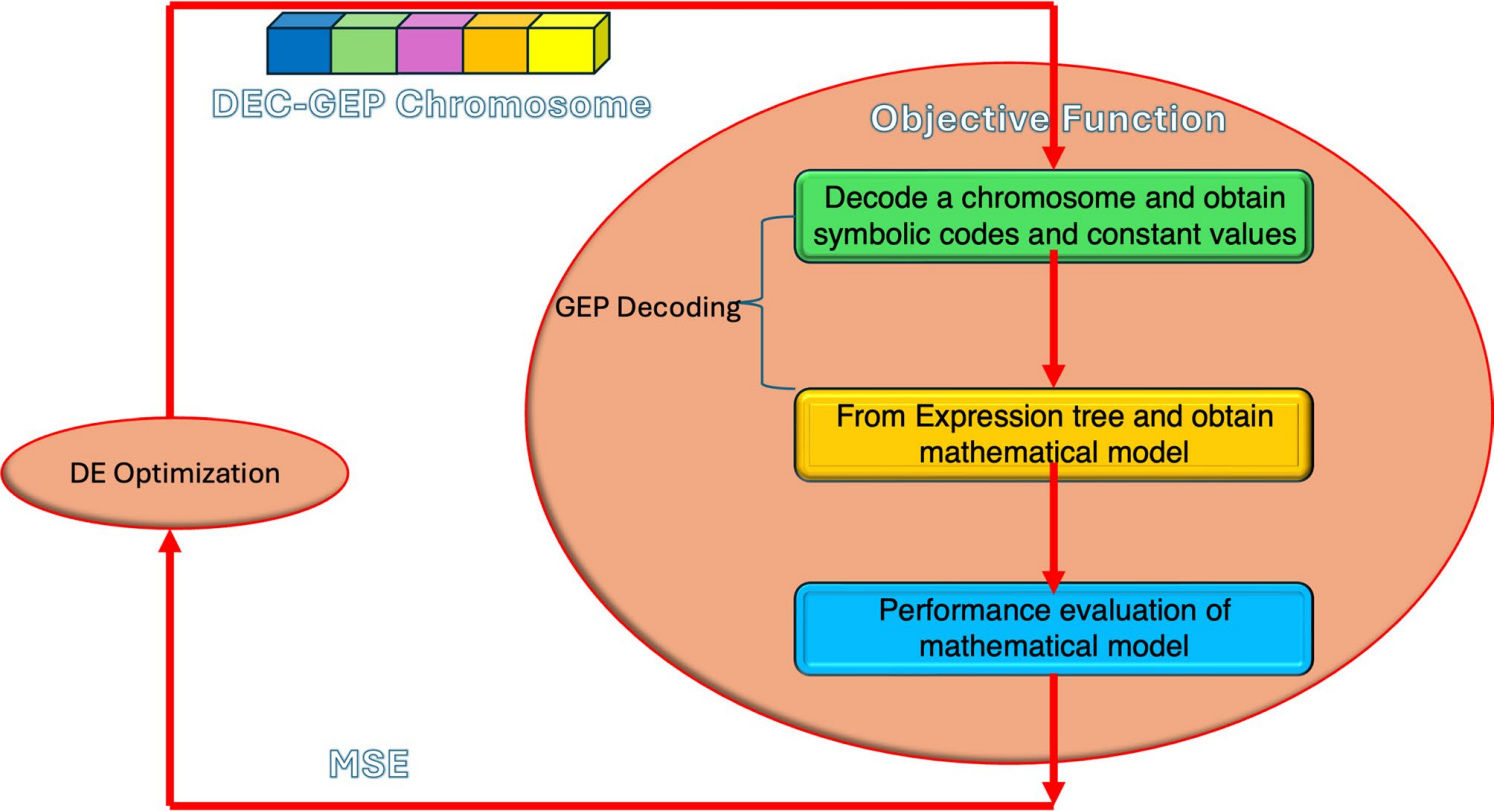
A flowchart of the DEC-GEP algorithm.

The DEC-GEP algorithm flowchart depicted in Fig. 17 demonstrates how the DE algorithm interacts with GEP. The objective function within the DE algorithm operates in two main stages: it first decodes GEP chromosomes into valid mathematical expressions, then evaluates these expressions for performance. The DE algorithm works to enhance chromosome codes, while the objective function produces the Mean Squared Error (MSE)^41^ to measure how accurately the derived mathematical expressions match the training data.

To identify the best equation, several rounds of testing and detailed analysis of each potential equation are necessary. The evaluation process includes testing various custom functions along with an extensive range of operators and multiple combinations of dimensionless input variables. Each equation produced undergoes thorough evaluation and refinement to achieve a clear and precise representation that maintains accuracy. This study ensures equations balance complexity and the dual needs of precision and clarity (Note: These equations are usable for this study, not in general).

One limitation of the present study is the database size, which comprises 27 flexural and 27 compression FEM-generated data points. Although these data were carefully generated using validated finite element models, a limited dataset may limit the generalizability of the derived equation.

In this study, Gene Expression Programming was used to evolve a mathematical equation that best represents the relationship between the selected parameters and the structural response. The quality of the generated model was evaluated using statistical performance metrics such as the coefficient of determination (R²) and mean squared error (MSE). These indicators measure the agreement between the predicted results from the GEP equation and the FEM simulation outputs.

Because GEP generates an explicit analytical expression, the resulting equation can be directly compared with the available FEM data. Nevertheless, the dataset’s limited size may introduce uncertainty in the model’s predictive performance on unseen configurations. Adding additional FEM simulations or experimental data to the database would further improve the model’s robustness.

Unlike traditional regression approaches that assume a predefined functional form, GEP performs symbolic regression, where mathematical expressions evolve through evolutionary operations such as mutation, recombination, and selection. As a result, the final equation generated by the algorithm can capture complex nonlinear relationships between input parameters and structural response.

Therefore, nonlinear interactions among the selected variables were implicitly accounted for during the evolutionary optimization process, enabling the derived equation to capture the nonlinear behavior observed in the finite element simulations.

A systematic procedure enables researchers to identify optimal expressions that define transverse compressive and flexural capacity, which are shown as:

1 $$\:{Normalized}_{Ccap}=\frac{T+T\times\:{f}_{c}^{{\prime\:}}}{T{\times\:P}_{d}+T+1}\:$$

2 $$\:Ccap={Min}_{Ccap}+{Normalized}_{Ccap}\times\:({Max}_{Ccap}-{Min}_{Ccap})$$

3 $$\:Ccap=71+\frac{T+T\times\:{f}_{c}^{{\prime\:}}}{T{\times\:P}_{d}+T+1}\times\:449.1$$

Where $\:T$ represents the thickness of the concrete element, and $\:{f}_{c}^{{\prime\:}}$​ denotes the concrete compressive strength. The term $\:{P}_{d}$​ corresponds to the perforation diameter. $\:{Normalized}_{Ccap}$ refers to the normalized transverse compression capacity, which is a dimensionless value scaled between 0 and 1. $\:Ccap$​ represents the actual transverse compression capacity. In this context, $\:{Max}_{Ccap}\:\:and\:{Min}_{Ccap}\:$are the maximum and minimum transverse compression capacities, respectively, used to rescale the normalized result to its original range.

4 $$\:{Normalized}_{Fcap}=\frac{T+{f}_{c}^{{\prime\:}}}{{P}_{d}^{3}+{P}_{d}^{2}+2}\:$$

5 $$\:Fcap={Min}_{Fcap}+{Normalized}_{Fcap}\times\:({Max}_{Fcap}-{Min}_{Fcap})$$

6 $$\:Fcap=113.8+\frac{T+{f}_{c}^{{\prime\:}}}{{P}_{d}^{3}+{P}_{d}^{2}+2}\times\:670.6$$

Where $\:{Normalized}_{Fcap}$ refers to the normalized flexural capacity, which is a dimensionless value scaled between 0 and 1. $\:Fcap$​ represents the actual flexural capacity. In this context, $\:{Max}_{Fcap}\:\:and\:{Min}_{Fcap}\:$are the maximum and minimum flexural capacities, respectively, used to rescale the normalized result to its original range.

**Fig. 18:**
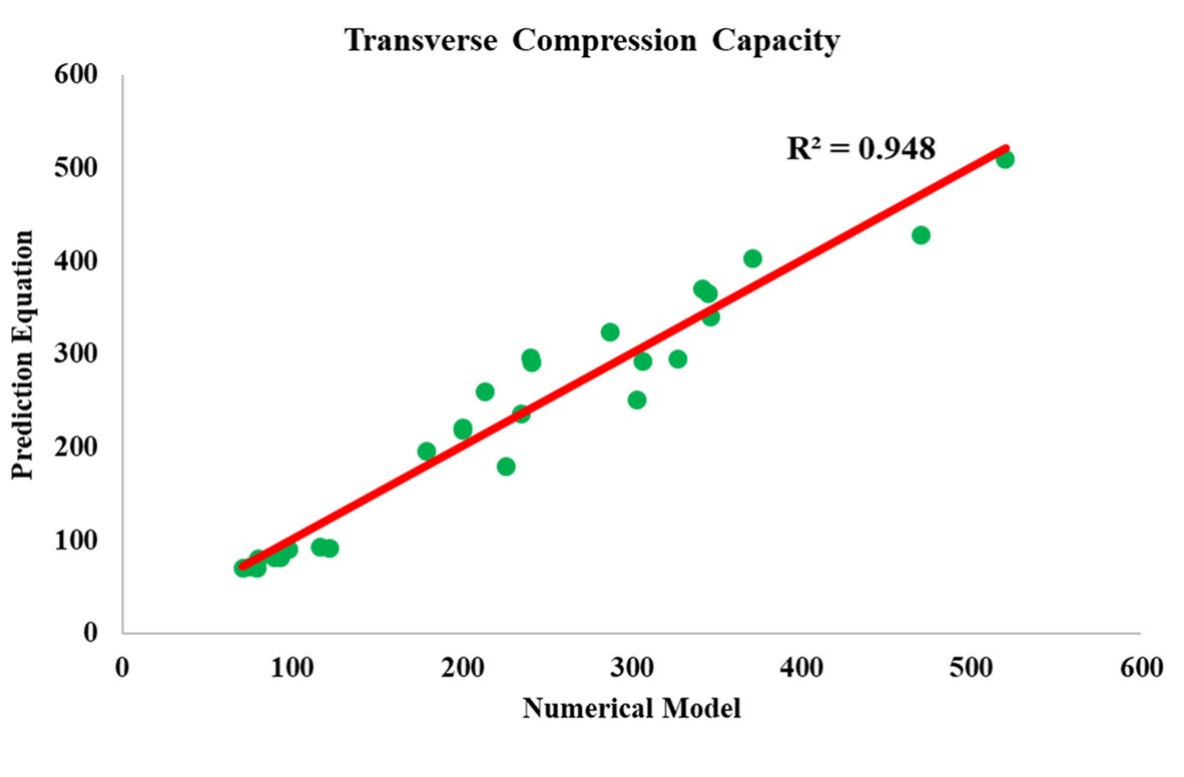
Predicted vs. numerical transverse compression capacity.

**Fig. 19:**
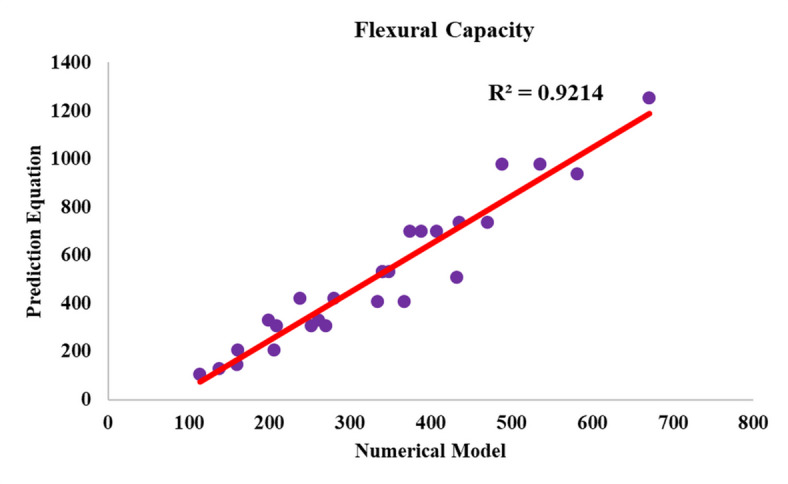
Predicted vs. numerical flexural capacity.

Figures 18 and 19 demonstrate a strong relationship between the developed predictive equations and numerical model results for flexural and transverse compression capacities, using Eqs. (3) and (6). The model achieves a coefficient of determination ($\:{R\:}^{2}$) of 0.9214 in the first graph related to flexural capacity, highlighting its accurate prediction of numerical outputs. The second graph shows the predictions for transverse compression capacity, which achieves a value of $\:{R\:}^{2}$= 0.948, indicating excellent agreement between the prediction equation and the numerical model. The proposed equations demonstrate their reliability and effectiveness through accurate estimates of structural capacity.

## Applicability and limitations of the proposed equations

The equations obtained using DEC-GEP are empirical predictive expressions derived from the available dataset. Therefore, their applicability is limited to the parameter ranges and geometric configurations considered in this study.

The proposed equations should only be used within:

- The range of geometric parameters included in the FEM simulations.
- The material properties considered in the study.
- The loading and boundary conditions used in the numerical models.

Applying the equations outside these ranges may yield inaccurate predictions because the model has not been trained under those conditions. Therefore, extrapolation beyond the studied parameter space should be performed with caution.

Future research incorporating larger datasets and additional structural configurations may extend the applicability of the developed equations.

## Conclusions

This study investigated the structural performance of hexagonal, hollow, perforated SEAHIVE^®^ concrete units reinforced with GFRP bars under transverse compression and flexural loading. The primary objective was to establish a validated structural capacity framework and develop design-oriented predictive equations by integrating experimental testing, nonlinear finite element modeling, and artificial intelligence.

The results demonstrate that both geometric and material parameters significantly influence structural capacity. Increasing concrete compressive strength from 20 MPa to 40 MPa enhanced transverse compressive capacity by up to 60% and flexural capacity by up to 66%. Increasing element thickness from 100 mm to 180 mm produced the most pronounced effect, resulting in more than a 280% improvement in transverse compressive resistance and substantial gains in flexural capacity due to increased cross-sectional stiffness. In contrast, larger perforation diameters intensified stress concentrations and generally reduced capacity. Overall, thickness was identified as the dominant parameter governing compression-dominated behavior, whereas both thickness and concrete strength played major roles in flexural response.

The nonlinear ABAQUS models employing the concrete damage plasticity (CDP) formulation successfully reproduced experimental load–displacement curves and crack propagation patterns, confirming the robustness of the modeling approach. This experimentally validated FEM framework enabled systematic quantification of interacting geometric and material effects in perforated hollow systems where classical analytical methods are inadequate.

To enhance practical applicability, Differential Evolution Chromosomal Gene Expression Programming (DEC-GEP) was used to derive explicit predictive equations for transverse compression and flexural capacities. The resulting symbolic regression models achieved coefficients of determination (R²) of 0.948 and 0.921, respectively, demonstrating strong predictive capability while maintaining interpretability suitable for engineering design. The developed FEM framework and AI-based equations provide engineers with a practical tool for rapid estimation of SEAHIVE^®^ structural performance during preliminary design, reducing the need for extensive numerical simulations.

Collectively, the integrated experimental–numerical–AI framework addresses a critical gap in the structural evaluation of perforated, hollow, GFRP-reinforced coastal units and provides engineers with validated tools for preliminary design and optimization. The FEM framework may be employed to assess alternative configurations prior to fabrication, while the derived equations enable rapid parametric screening during conceptual design stages.

Nevertheless, the predictive equations are valid only within the investigated parameter ranges and geometric configuration. Future research should extend the database to include additional geometries, reinforcement ratios, cyclic and dynamic loading conditions, durability effects, and full-scale validation. Such efforts will further enhance the generalizability and practical implementation of perforated coastal structural systems.

## Data Availability

Data will be made available upon request from the corresponding author.
